# Pilot Production of Mesenchymal Stem/Stromal Freeze-Dried Secretome for Cell-Free Regenerative Nanomedicine: A Validated GMP-Compliant Process

**DOI:** 10.3390/cells7110190

**Published:** 2018-10-30

**Authors:** Elia Bari, Sara Perteghella, Dario Di Silvestre, Marzio Sorlini, Laura Catenacci, Milena Sorrenti, Giorgio Marrubini, Rossana Rossi, Giuseppe Tripodo, Pierluigi Mauri, Mario Marazzi, Maria Luisa Torre

**Affiliations:** 1 Department of Drug Sciences, University of Pavia, Viale Taramelli 12, 27100 Pavia, Italy; elia.bari@unipv.it (E.B.); laura.catenacci@unipv.it (L.C.); milena.sorrenti@unipv.it (M.S.); giorgio.marrubini@unipv.it (G.M.); giuseppe.tripodo@unipv.it (G.T.); marina.torre@unipv.it (M.L.T.); 2 PharmaExceed srl, 27100 Pavia, Italy; marzio.sorlini@supsi.ch; 3 Institute for Biomedical Technologies, F.lli Cervi 93, 20090 Segrate, Milan, Italy; dario.disilvestre@itb.cnr.it (D.D.S.); rossana.rossi@itb.cnr.it (R.R.); pierluigi.mauri@itb.cnr.it (P.M.); 4 Department of Innovative Technologies, University of Applied Sciences and Arts of Southern Switzerland, SUPSI, Via Pobiette 11, 6928 Manno, Switzerland; 5 Tissue Therapy Unit, ASST Niguarda Hospital, Piazza Ospedale Maggiore 3, 20162 Milan, Italy; mario.marazzi@ospedaleniguarda.it

**Keywords:** exosome, microvesicles, ultrafiltration, freeze-drying, secretome, mesenchymal stem cells

## Abstract

In this paper, a pilot production process for mesenchymal stem/stromal freeze-dried secretome was performed in a validated good manufacturing practice (GMP)-compliant cell factory. Secretome was purified from culture supernatants by ultrafiltration, added to cryoprotectant, lyophilized and characterized. We obtained a freeze-dried, “ready-off-the-shelf” and free soluble powder containing extracellular vesicles and proteins. In the freeze-dried product, a not-aggregated population of extracellular vesicles was detected by nanoparticle tracking analysis; Fourier transform infrared spectra showed the simultaneous presence of protein and lipids, while differential scanning calorimetry demonstrated that lyophilization process successfully occurred. A proteomic characterization allowed the identification of proteins involved in immune response, response to stress, cytoskeleton and metabolism. Moreover, the product was not cytotoxic up to concentrations of 25 mg/mL (on human fibroblasts, chondrocytes and nucleus pulposus cells by MTT assay) and was blood compatible up to 150 mg/mL. Finally, at concentrations between 5 and 50 mg/mL, freeze-dried secretome showed to in vitro counteract the oxidative stress damage induced by H_2_O_2_ on nucleus pulposus cells by MTT assay.

## 1. Introduction

Mesenchymal stem/stromal cells (MSCs) currently represent an effective tool for the treatment of various diseases, thanks to their tissue protective and reparative mechanisms [1,2]. MSC therapeutic effectiveness has been proved by almost 810 worldwide clinical trials (http://clinicaltrials.gov, last search: 31 March 2018), with a plethora of diseases treated [3]. Initially, it was believed that their therapeutic effects derived from MSC migration into damaged tissue and subsequent differentiation to replace dead cells. This mechanism of action was re-envisioned at the beginning of the twenty-first century when, for the first time, Gnecchi and colleagues showed that MSCs mediate their therapeutic effects by the release of paracrine factors (known as secretome) [4].

MSC-secretome is composed of a heterogeneous pool of bioactive molecules secreted both as free soluble factors (including cytokines, chemokines and growth factors) and as insoluble nano/microstructured-vesicles, known as extracellular vesicles (EVs) [5]. In addition, to be involved in intercellular communication, the secretome mirrors the genetic and proteomic content of the secreting MSCs, and thus is assumed to be able to reproduce the therapeutic effects of stem cells themselves [6,7,8]. Several authors employed so-called conditioned media (CM) (which is made by the culture medium, including all the bioactive molecules released by MSCs) and showed its effectiveness in enhancing bone regeneration in rat calvarial bone defects [9], in inducing apoptosis in human glioma cell lines in vitro [10], or in improving the quality of healed skin in chronic excisional wounds in rats [11]. MSC-secretome also showed a protective effect on cardiomyocytes [12], efficacy in preventing diabetic renal injury [13], and in sustaining tissue repair and regeneration on both acute or chronic wound models [14]. Again, MSC-EVs activate Vascular Endotelial Growth Factor (VEGF) receptors and the angiogenesis pathways accelerating recovery of hindlimb ischemia [15], promote collagen I and elastin synthesis in the skin, which are essential to skin rejuvenation [16], and improve erectile function in induced diabetic rats [17].

In addition, MSC-EVs represent not only a potential active pharmaceutical ingredient (API), but also have promising characteristics to be used as drug delivery systems, mainly as a result of their homing ability, which allows a specific and targeted drug release into a damaged lesion or tumor. In this context, our research group combined biological-technological approaches and developed an innovative carrier-in-carrier system based on stem cell EVs that internalized silk/curcumin nanoparticles [18]. This innovative nanosystem combines beneficial effects of both regenerative cell therapies and pharmaceutical nanomedicine, avoiding the use of viable replicating stem cells. However, the limitations regarding EVs large-scale isolation, standardization and characterization still remain.

First of all, many different procedures to isolate whole secretome or its EV fraction have been described in the literature. Unfortunately, a standardized isolation process is still lacking, generating uncertainty about secretome/EVs biological effects, which seem strongly influenced by the preparation method [19,20,21]. A key aspect is the secretome isolation from cell culture supernatants by a validated, scalable and GMP-compliant production process, which allows obtaining standardized products suitable for clinical applications. Several isolation methods are reported in literature, such as size-exclusion chromatography (SEC) or the widely-used ultracentrifugation (UC); unfortunately, they are not scalable and, related to UC, an EV aggregation or breakup may occur due to the high shear forces. Subsequently, a secretome full characterization is required to confirm the reproducibility of the isolation method and in view of regulatory requirements.

Based on these premises, here we present an innovative GMP-compliant procedure for the large-scale production of lyo-secretome, a lyophilized ready-to-use product, based on adipose MSC-secretome, suitable to replace stem cells in regenerative nanomedicine. In particular, two common pharmaceutical techniques, ultrafiltration and freeze-drying, have been positively combined for large-scale lyo-secretome production under controlled conditions. As a quality control, a physico-chemical characterization of this new product was performed, as well as its molecular characterization at the proteomic level. Moreover, cytotoxicity on human fibroblasts (HF), chondrocytes (HC) and nucleus pulposus (NP) cells was performed, blood compatibility was assessed for safety requirement, and antioxidant properties were in vitro tested on NP cells as a highly responding model cell to oxidative stress.

## 2. Materials and Methods

Culture media, trypsin and antibiotics used for cell cultures were purchased from Euroclone (Milan, Italy). Collagenase, mannitol, bovine serum albumin, Nile Red, acetone, phosphatidylcholine, DPPH, were obtained from Sigma-Aldrich (Milan, Italy). A commercial platelet lysate kit (PL) was obtained from Carlo Erba Reagents (Milan, Italy). Collagenase and FBS were suitable for the production of clinical-grade human MSCs.

### 2.1. Lyo-Secretome Production Process in a GMP-Compliant Cell Factory

**Figure 1:**
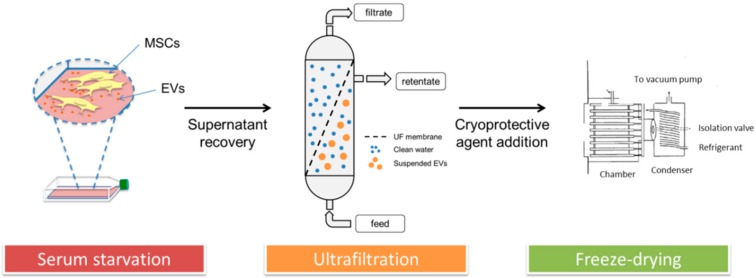
Lyo-secretome production process.

Three different lyo-secretome batches were produced from adipose-derived mesenchymal stromal cells (ASCs) harvested from 3 different informed donors, through a GMP-compliant process at the Tissue Therapy Unit, Niguarda Hospital (Milan, Italy). The study was conducted in accordance with the Declaration of Helsinki, and the protocol was approved by the Ethics Committee of ASST Grande Ospedale Metropolitano Niguarda (Milan, Italy) (Ref. 12 November 2009). In detail, the lyo-secretome production was performed according to the patented procedure reported by Perteghella et al. (see flowchart in Figure 1) [22]. Briefly, secretome was collected with cell culture supernatants, purified by ultrafiltration, added to cryoprotectant and freeze-dried, obtaining lyo-secretome. All the production steps were performed complying with the principles of GMP, through the knowledge of all critical steps for all the production phases to ensure a final product with the expected quality. Specifically, all the procedures were performed in aseptic conditions in a grade B cleanroom suite by qualified operators equipped with protective and disposable clothes; raw materials were introduced into the production room separately through a clean pass-box. Both the cell culture and lyo-secretome processes followed a qualification/validation protocol, also considering instruments, supplies and reagents, and defining roles and responsibilities for each step. Finally, as quality control analysis and GMP batch release testing are part of the manufacturing process, on the finished product, in view of regulatory requirements concerning quality, safety and efficacy, a characterization was performed.

#### 2.1.1. Isolation and Expansion of Human Adipose-Derived Mesenchymal Stromal Cells

Adipose tissues were obtained from 3 patients (mean age was 47 ± 4) undergoing abdominoplasty surgery after informed consent (ASST Grande Ospedale Metropolitano Niguarda, Milan). Clinical sheets of donors (identity, gender, age, tissue processing, sampling site, the day of collection and anamnesis) were filed by the surgery-responsible structure. Donors with septicemia or extensive infections, syphilis, type B and C hepatitis, HIV, Creutzfeld-Jacobs disease, viral or unknown neurological diseases, human GH treatment, and malignant tumors were excluded from the trial. Tissue samples were repeatedly washed with Phosphate Buffer Saline (PBS, pH = 7.14) without Ca^2+^ and Mg^2+^ and mechanically minced by surgical scissors. Tissue digestion was performed with 0.075% (*w*/*v*) type II collagenase solubilized in PBS with Ca^2+^ and Mg^2+^ plus 1% penicillin/streptomycin and 1% (*w*/*v*) amphotericin B at 37 °C. After 1 h, Dulbecco’s modified Eagle’s Medium (DMEM) and Ham’s medium F12 (DMEM/F12, ratio 1:1) supplemented by 10% (*v*/*v*) fetal bovine serum (FBS) was added to cell suspension; the digested tissue was filtered on a 70 µm cell strainer (Greiner Bio-One, Milan, Italy) and centrifuged at 600× *g* for 5 min [23,24]. Recovered stromal vascular fraction was cultured in monolayer conditions (100,000 cells/cm^2^) in DMEM F12, 10% (*v*/*v*) FBS, 1% (*v*/*v*) penicillin/streptomycin and 1% (*v*/*v*) amphotericin B. Once MSCs reached sub-confluence, they were treated with 0.05% (*v*/*v*) trypsin-EDTA and seeded onto flasks (10,000 cells/cm^2^) at 37 °C and 5% CO_2_ and cultured until P6 in DMEM F12, 5% (*v*/*v*) PL, 1% (*v*/*v*) penicillin/streptomycin and 1% (*v*/*v*) amphotericin B. All MSCs were tested to ensure concordance with all the requirements needed for clinical use in terms of identity (according to the International Society for Cellular Therapy), sterility (according to Eu. Ph. 9.0, 2.6.27), tumorigenesis and karyotype.

#### 2.1.2. MSC-Secretome Collection

Briefly, to induce secretome release, once reaching sub-confluence, MSCs were cultured in serum-free medium [18,25] for 24 h, replaced with fresh medium after 9 h. At the end of the experiment, conditioned media collected at 9 and 24 h were mixed; MSCs were instead detached with trypsin-EDTA to evaluate cell viability by iodide propidium exclusion cytofluorimetric analysis validated according to ICH Q2 guidelines.

#### 2.1.3. MSC-Secretome Purification: Ultrafiltration

Conditioned media were at first centrifuged at 3500× *g* for 10 min to eliminate cell debris and apoptotic bodies. Then, supernatants were collected and MSC-secretome purification was performed by tangential flow filtration using KrosFlo^®^ Research 2i system (Spectrum Laboratories, Milan, Italy), equipped by a 5 kDa Molecular Weight Cut Off (MWCO) filtration module (Spectrum Laboratories, Milan, Italy). All parts of the instrument were sterilized before use and ultrafiltration was conducted in aseptic conditions under a laminar flow hood in a B cleanroom suite. The automated process allowed, first, concentration, and, then, diafiltration of samples; according to manufacturer’s instruction, during both steps, the shear rate of the feed stream was maintained between 2000 s^−1^ and 6000 s^−1^, while trans-membrane pressure did not exceed 5 psi. The concentration step was stopped when a concentration of 0.5 × 10^6^ cell equivalents per mL was reached. For the diafiltration step, sterilized ultrapure water was used. To evaluate the industrial process scalability, average liters per m^2^ per h was calculated as follows:

L/m^2^/h = permeate flux (mL/min)/cartridge superficial area (m^2^) × 0.06 (1)

#### 2.1.4. Secretome Freeze-Drying (FD)

Mannitol was chosen as a cryoprotectant and dissolved into purified secretome to obtain the final concentration of 0.5% (*w*/*v*). The resulting solution was frozen at −80 °C and freeze-dried (Christ Epsilon 2-16D LSCplus) at 8 × 10^−1^ mbar and −50 °C for 72 h. The obtained lyo-secretome was stored at −20 °C until use. At the end of the lyophilization process, lyo-secretome yield was determined and, for each batch, cell equivalents per mg were calculated dividing the total cell number used for production and the obtained milligrams of lyo-secretome.

#### 2.1.5. Microbiological Control

The sterility of the final product was assured by the employment of materials with a proved sterility validation document and by microbiological controls on the resulting output [26]. A control of all the reagents used was performed before the purification process (t_0_). At each production step, representative samples were collected, suspended in a culture medium and stored for 7 d in a temperature-mapped, validated incubator at 37 °C. After 1 d (t_1_) and 7 d (t_2_) a microbiological test was performed in a GMP-certificated laboratory.

### 2.2. Lyo-Secretome Characterization

#### 2.2.1. Determination of Total Protein Content

Lyo-secretome total protein content was determined at the end of the lyophilization process using the micro BCA-Protein Assay Kit (Thermo Fischer Scientific, Milan, Italy) following the manufacturer’s specifications, after sample re-suspension in deionized water. The absorbance–concentration calibration curve was generated using bovine serum albumin (BSA) standards. Working reagent solution was added to each sample or standard (ratio 1:1) and then incubated at 37 °C for 2 h before measuring the absorbance at 562 nm with a microplate reader (Synergy HT, Milan, Italy). The concentration of unknown lyo-secretome protein content was computed from a plot of concentration vs. absorbance obtained for the standard protein solutions, using a third-order polynomial equation, with R^2^ = 0.99. Each sample was tested in triplicate; results are reported as µg of protein per mg of lyo-secretome.

#### 2.2.2. Phospholipid Quantification by Nile Red Assay

Nile Red powder was solubilized in acetone (3.14 M) and stored at 4 °C, avoiding light exposure. The stock solution was diluted 100× in PBS (pH = 7.14) before use. A quantity of 90 µL of dissolved lyo-secretome were incubated with 10 µL of diluted Nile Red solution for 5 min and the relative fluorescence was measured by Synergy HT at fixed wavelengths (530/25 excitation and 645/40 emission). The fluorescence–concentration calibration curve was generated using phosphatidylcholine (PC) standards, with R^2^ = 0.99. Each sample was tested in triplicate; results are reported as µg of lipid per mg of lyo-secretome.

#### 2.2.3. BCA-Protein Assay and Nile Red Assay Validation

To meet the pharmaceutical quality requirements, both BCA-Protein and Nile Red assays were validated. In detail, the parameters studied for method validation were specificity, linearity, range, accuracy and precision (expressed by repeatability and intermediate precision). The raw data are reported in the Supplementary Materials.

#### 2.2.4. EV Particle Size Distribution

Lyo-secretome particle size distribution was performed by nanoparticle tracking analysis (NTA) using NanoSight NS 300 equipment (Malvern Instruments, Malvern, UK). The semi-milled product was analyzed as such, while freeze-dried powder was first dispersed in deionized water (1 mg/mL) and analyzed.

#### 2.2.5. Ultrastructure Investigation by Transmission Electron Microscopy (TEM) and Morphology Investigation by Scanning Electron Microscopy (SEM)

At the end of the ultrafiltration process, EV ultrastructure was investigated by TEM with a JEOL JEM 1200 EX instrument. To provide a tridimensional scaffold in which the EV fraction of secretome was immobilized, an alginate matrix was prepared according to previously reported procedures [18,27]. Briefly, medium viscosity alginate was solubilized in physiological saline solution (2%, *w*/*v*) and sterilized by filtration. Purified secretome was added to the alginate solution (volume ratio 1:3) and extruded drop by drop through a 22G-needle into an isotonic saline solution containing 50 mM CaCl_2_ under magnetic stirring. As a consequence of the calcium ion exchange, alginate beads were formed. Encapsulated EV fractions were fixed in a solution of 2.5% paraformaldehyde and 2% glutaraldehyde, followed by 1% osmium tetroxide, embedded in Epon812/Araldite mixture and processed for TEM. Consecutive ultrathin sections were stained with uranyl acetate-lead citrate for conventional TEM or underwent immune-gold staining with the HSP90 monoclonal antibody, to detect heat shock protein-90 β, which is expressed at the surface of mesenchymal EVs [28].

After lyophilization, lyo-secretome morphology was investigated by SEM with a Zeiss EVO MA10 (Carl Zeiss, Oberkochen, Germany). The samples were gold-sputter coated under argon before performing the analysis.

#### 2.2.6. Fourier Transform Infrared Spectroscopy (FT-IR)

FT-IR analysis was performed to confirm the simultaneous presence of both soluble proteins and EVs into lyo-secretome. FT-IR spectra were collected using a Spectrum One Perkin-Elmer spectrophotometer equipped with a MIRacle™ ATR device. The IR spectra in transmittance mode were obtained in the spectral region of 650–4000 cm^−1^ by the accumulation of 64 scans with a resolution of 4 cm^−1^.

#### 2.2.7. Thermal Characterization

Differential scanning calorimetry (DSC) analysis was performed with a Mettler STARe system equipped with a DSC821e Module and an Intracooler device (Julabo FT 900) for sub-ambient temperature analysis. The curves were recorded on about 3 mg of sample in 40 µL sealed aluminum pans with pierced lid (method: −30–250 °C temperature range; heating rate 10 K min^−1^; nitrogen air atmosphere flux 50 mL min^−1^). The instrument was previously calibrated with indium, as a standard reference. Each experiment was performed in triplicate. Thermo gravimetric analysis (TGA) was performed with a Mettler STARe system equipped with a TGA/DSC1. The curves were recorded on about 4 mg of samples in 70 µL alumina pans (method: 30–300 °C temperature range; heating rate 10 K min^−1^; nitrogen air atmosphere flux 50 mL min^−1^). The instrument was previously calibrated with indium, as a standard reference. Each experiment was performed in triplicate.

To simulate the lyophilization process, the DSC curves were recorded on about 30 mg of solution in 100 µL sealed aluminum pans with lid with a method that provides a first cooling step between 30 and −60 °C, an isothermal step at −60 °C, followed by a heating step between −60 and 60 °C, with a heating rate of 2 K min^−1^.

#### 2.2.8. Sample Preparation for Proteomic Analysis

The sample collected at the end of ultrafiltration and before cryoprotectant addition (1 mL) were concentrated to 50 µL using a SpeedVac system (Savant Instruments, Farmingdale, NY, USA) and treated with RapiGest SF (Waters Corporation, Milford, MA, USA) at the final concentration of 0.25% (*w*/*v*). After incubation at 100 °C for 20 min, the samples were cooled at room temperature and centrifuged at 2200× *g* for 10 min. Subsequently, protein concentration was assayed using the SPNTM Protein Assay kit (G-Biosciences, St. Louis, MO, USA) and 50 ± 0.5 μg of protein from each sample was digested with Sequencing Grade Modified Trypsin (Promega, Madison, WI, USA) using a 1:50 (*w*/*w*) enzyme/substrate ratio at 37 °C overnight. The next morning, an additional aliquot of enzyme was added (enzyme/substrate ratio of 1:100 (*w*/*w*)). The enzymatic reaction was chemically stopped after 4 h by acidification with TFA 0.5% (Sigma-Aldrich Inc., St. Louis, MO, USA), incubation at 37 °C for 45 min and centrifugation at 13,000× *g* for 10 min in order to remove hydrolytic RapiGest SF by-products. Finally, the samples were desalted by PierceTM C-18 spin columns (Thermo Fisher Scientific, Waltham, MA, USA), concentrated in a SpeedVac (Savant Instruments, Farmingdale, NY, USA) at 60 °C and resuspended in 0.1% formic acid (Sigma-Aldrich Inc., St. Louis, MO, USA) at a concentration of 0.1 µg/µL.

#### 2.2.9. LC-MS/MS Analysis

Trypsin-digested proteins were analyzed by the Eksigent nanoLC-Ultra 2D System (Eksigent, AB SCIEX, Dublin, CA, USA) configured in trap-elute mode. Briefly, for each sample, a total of 0.8 µg digested proteins were first loaded on the nanoLC trap (350 μm × 500 μm ChromXP C18, 3 μm, 120 Å) and washed in isocratic mode with 0.1% aqueous formic acid for 10 min at a flow rate of 3 μL/min. The automatic switching of nanoLC ten-port valve then eluted the trapped mixture on a nano LC column (75 μm × 15 cm 3C18-CL, 3 μm, 120 Å), through a 75 min gradient of 5–45% of eluent B (eluent A, 0.1% formic acid in water; eluent B, 0.1% formic acid in acetonitrile), at a flow rate of 300 nL/min. Mass spectra were acquired using a LTQ-Orbitrap XL-ETD mass spectrometer (Thermo Fisher Scientific, San José, CA, USA), equipped with a nanospray ionization source (Thermo Fisher). Nanospray was achieved using a coated fused silica emitter (New Objective, Woburn, MA, USA) (360 μm o.d./50 μm i.d.; 730 μm tip i.d.) held at 1.6 kV. The ion transfer capillary was held at 220 °C. Full mass spectra were recorded in positive ion mode over a 400–1600 *m*/*z* range and with a resolution setting of 30,000 Full Width at Half Maximum (FWHM) and scan rate of 2 spectra per s, followed by 5 low-resolution MS/MS events, sequentially generated in a data-dependent manner on the top 5 most intense ions selected from the full MS spectrum, using dynamic exclusion for MS/MS analysis. In particular, MS/MS scans were acquired setting a normalized collision energy of 35% on the precursor ion and, when a peptide ion was analyzed twice, applying an exclusion duration of 0.5 min.

#### 2.2.10. MS/MS Data Processing

The experimental MS/MS spectra were matched against the in silico tryptic peptide sequences of the *homo sapiens* protein database (71,599 protein sequences) retrieved from UNIPROT database on May 2018. Data processing was performed by Discoverer 2.1 software, based on SEQUEST HT algorithm [29]. Peptide and protein assignment was made according to specific guidelines [30]. The following criteria were used for peptide identification (Figure S1): specifically, parent and fragment mass tolerance of 0.6 Da and 50 ppm, respectively, and missed cleavage sites per peptide of 2. Matches between spectra were only retained if they had a minimum Xcorr of 2.0 for +1, 2.5 for +2, and 3.5 for +3 charge state, respectively, protein rank was fixed to 1, while peptide confidence was set to “high”. In addition, the false discovery rate (FDR) was set to ≤1%. The average SpC (aSpC) values of the identified proteins were calculated selected by MAProMa software. Functional Annotation Tool of DAVID database [31] was used to characterize the most enriched cellular component (CC), molecular function (MF) and biological processes (BP). Specifically, a count >5 and *p* < 0.001 were set. Finally, a protein-protein interaction (PPI) network was built by combining proteins identified by analyzing the secretome of human adipose-derived mesenchymal stromal cells and interactomic data retrieved from the major repositories. In addition, it was integrated by in-house manually curated data derived from literature. The resulting sub-networks were visualized and analyzed by Cytoscape and its plugins [32]. In particular, Cytoscape plugin BINGO 2.44 [33] was used for evaluating the most represented GO terms; *homo sapiens* organism, hypergeometric test, Benjamini–Hochberg FDR correction and a significance level ≤0.001 were applied.

### 2.3. Lyo-Secretome Safety Assays

#### 2.3.1. Blood Compatibility

Blood compatibility was assessed by hematolytic and hemagglutination tests after lyo-secretome re-suspension at different concentrations (25, 50, 75, 100 and 150 mg/mL). Fresh human blood samples were collected from at least three consent-informed healthy individual donors (female blood). In detail, the hemolytic assay was performed as reported by Maji et al. with some modifications [34]. Red blood cells (RBCs) were separated from plasma by centrifugation at 1500× *g* for 5 min. The RBCs pellet was washed twice with PBS without Ca^2+^ and Mg^2+^ (pH = 7.4). 90 µL of RBCs suspension were co-incubated with 10 µL of lyo-secretome, re-suspended in PBS at the previously reported concentrations, and incubated at room temperature for 60 min. RBCs treated with PBS were considered as the negative control, while RBCs treated with distilled water were considered as the positive control (100% of hemolysis). After incubation, samples were centrifuged at 3000× *g* for 10 min and, on the recovered supernatants, optical density (OD) was measured at 540 nm (NanoPhotometer NP80 IMPLEM), which corresponds to the maximum absorption of hemoglobin. Hemolysis (%) was evaluated with the following equation:

Hemolysis (%) = (OD sample − OD blank)/(OD positive CTRL) × 100(2)

where OD sample is the absorbance of samples, OD blank is the absorbance of the negative control and OD positive CTRL is the absorbance of RBCs treated with distilled water.

Hemagglutination activity of lyo-secretome was evaluated following the procedure reported by [35,36] with slight modifications. Briefly, 100 μL of the RBCs pellet was mixed with 10 mL of PBS (pH 7.4) and used in the assay. In a 96-well ‘U-shaped’ plate, 50 μL of each lyo-secretome concentrations were added to 50 μL of RBCs suspension in each well; PBS was used as negative control. All the samples were incubated at 37 °C for 2 h and the formation of ‘button like’ structure was evaluated.

#### 2.3.2. Cytocompatibility Test

Lyo-secretome cytocompatibility was evaluated on 3 different cell lines: human fibroblasts (HF), human chondrocytes (HC) and nucleus pulposus (NP) cells. HF, HC and NP cells were seeded in 96-well plate (10,000 cells/cm^2^) and cultured with their specific media (DMEM HG plus 10% fetal bovine serum, 100 U/mL penicillin, 100 μg/mL streptomycin, 0.25 μg/mL amphotericin, 4 mM glutamine, 1 mM sodium pyruvate for HF; DMEM HG plus 10% fetal bovine serum, 100 U/mL penicillin, 100 μg/mL streptomycin, 0.25 μg/mL amphotericin, Fibroblast Growth Factor-2, FGF-2, 10 µg/mL, and Transforming Growth Factor β1, TGF-β1, 1 µg/mL, for HC cells; DMEM HG plus 10% fetal bovine serum, 100 U/mL penicillin, 100 μg/mL streptomycin, 0.25 μg/mL amphotericin for NP cells). After 24 h, supernatants were discarded and replaced with 100 µL of culture medium in which lyo-secretome was solubilized at 3 different concentrations (5, 12.5 and 25 mg/mL). After 24 and 48 h of incubation, an MTT assay was performed and cell metabolic activity was calculated according to the following equation:

metabolic activity% = 100 × (ODs/ODc)(3)

where ODs is the mean value of the measured optical density of the tested samples and ODc is the mean value of the measured optical density of cells not incubated with lyo-secretome.

### 2.4. Lyo-Secretome Potency Assays

#### 2.4.1. ROS-Scavenging Activity Assay

The lyo-secretome ROS-scavenging activity was evaluated by the DPPH (2,2-diphenyl-2-picrylhydrazyl hydrate) method, according to previously published procedures, with slight modifications [37,38]. In detail, lyo-secretome was tested at different concentrations (25, 50, 100 and 200 mg/mL) after re-suspension in distilled water under magnetic stirring. 50 µL of each concentration were mixed with 1950 µL of DPPH solution (0.1 mM) in 70% *v*/*v* methanol and incubated in the dark for 20 min at room temperature. The OD was measured at 517 nm with a UV-Vis spectrophotometer. Ascorbic acid was used as positive control, while for negative control a reaction mixture composed of 50 µL of 70% *v*/*v* methanol and 1950 µL of DPPH solution was prepared. The ROS-scavenging activity was calculated according to the following equation:

ROS-scavenging activity% = (A − B)/A × 100(4)

where A is the OD of the negative control and B is the OD of the samples. Analyses were performed in 3 replicates, and results are reported as mean ± SD.

#### 2.4.2. Oxidative Stress Protection

The lyo-secretome ability to protect cells from oxidative stress was investigated on NP cells. NP cells were seeded in 96-well plate (10,000 cells/cm^2^). After 24 h, supernatants were replaced with 100 µL of culture media containing lyo-secretome, considering the following concentrations: 5, 25, 50, 75 and 100 mg/mL. After 24 h of incubation, media were discarded and 100 µL of hydrogen peroxide (1 mM) solution were added to each well. Cells not incubated with H_2_O_2_ were considered as control. After 24 h, for the cells incubated with or without H_2_O_2_, an MTT test was performed to evaluate the cellular metabolic activity, calculated as previously reported. All experiments were conducted in triplicate.

### 2.5. Statistical Analysis

Raw data were processed using STATGRAPHICS XVII (Statpoint Technologies, Inc., Warrenton, VA, USA). A linear generalized analysis of variance model (ANOVA) was used, followed by Fisher’s least significant difference (LSD) procedure to estimate the differences between means. The protein and lipid content of each batch was analyzed considering batch number as fixed factor and protein/lipid amount as the response variable. The cell cytotoxicity was analyzed taking into account the cell metabolic activity as the response variable and the lyo-secretome concentration and incubation time as fixed factors. The blood hemolysis was analyzed taking into account the hemolysis percentage as the response variable and the lyo-secretome concentration as a fixed factor. The DPPH assay results were processed considering the antioxidant activity percentage as the response variable and the lyo-secretome concentration as a fixed factor. In a similar way, the oxidative stress protection results were evaluated considering the cell metabolic activity as the response variable and the lyo-secretome concentration and presence/absence of H_2_O_2_ as fixed factors. Statistical significance was fixed at *p* < 0.05.

## 3. Results

### 3.1. Lyo-Secretome Production

The MSCs employed for the lyo-secretome production fulfilled all the requirements needed for clinical use, as reported in the literature, in terms of identity, tumorigenesis, karyotype and sterility [39]. Secretome from MSCs was obtained after 24 h in serum starvation conditions; after this incubation time, supernatants were collected, and cells were detached in order to determine their number and viability (Table 1).

The collected supernatants were at first concentrated to reduce volume and to obtain a fixed value of cell equivalents per mL (0.5 × 10^6^ cell). The filtration module chosen had a sufficiently low molecular weight that allows retaining not only the EVs but also the soluble proteins released by MSCs. Concentrated supernatants were then diafiltered at least five times with ultrapure deionized water to eliminate small and undesired compounds. On average, starting from about 500 mL of conditioned media, the whole process was concluded within 2 h using a filtration module with a superficial area of 115 cm^2^. For all the produced batches, the calculated ultrafiltration efficiency was 19.5 ± 3.99 L/m^2^/h (mean value ± standard deviation, *n* = 3) and the purification process was able to remove all the small and undesired compounds, including red phenol dye. Once purified, conditioned media were added to cryoprotectant and freeze-dried.

The performed microbiological test proved that sterility conditions were maintained during all the production steps as well as for both semi-finished and finished products, revealing the absence of mesophilic and psychrotrophic bacteria and fungi.

### 3.2. Lyo-Secretome Characterization

At the end of the lyophilization process, the obtained lyo-secretome was characterized in terms of total protein and lipid content; results are reported in Table 2 expressed as µg of protein/lipid per mg of lyo-secretome. To exclude contamination from the culture medium, the total proteins and lipids were also assessed into ultrafiltered and freeze-dried unconditioned media, without detecting their presence.

The yield is higher for batch 3, which was produced by a higher number of cells, and the amount of resulting protein and lipids is significantly different depending on the batch number. Moreover, protein content is not related to lipid content: batch 2 showed a higher protein content but not a higher lipid content, compared to batch 1. This indicates that cell line or cell density at the starvation time can strongly influence secretome production and composition.

It’s worth mentioning that both the methods used to determine the total protein and lipid content were validated to meet the pharmaceutical requirements. In detail, for BCA-protein assay, the mean coefficient of determination of the calibration curve computed in the range from 0 to 100 µg/mL of bovine serum albumin (BSA) in water on eleven model calibration curves after almost one year of application of the method was 0.982 ± 0.018. The curve equation, represented by the mean parameters ± confidence interval at 95% probability, had the equation y = (0.014 ± 0.003x + (0.3 ± 0.1) (Table S1). The intercept is not statistically significant, and the plot of the residuals showed a normal distribution of the error; both these observations showed that no systematic error affected the calibration results (data not shown). Method accuracy was demonstrated measuring samples at low (10 µg/mL), medium (30 µg/mL) and high (50 µg/mL) levels of BSA concentration, repeating the determinations three times for each level. The recovery percentages were always between 96 and 107%. The method intra-assay precision (repeatability) was assessed during one session of work measuring six independent samples at the level of concentration of 10, 30 and 50 µg/mL. The relative standard deviation percentage (RSD%) was lower than 5% for the series of measurements and, as such, it was considered a satisfactory value for the intra-assay precision. The next day, the samples were re-analyzed to assess the inter-assay precision (or intermediate precision). The data collected showed that the RSD% of the absorbance at levels of 50 and 30 µg/mL were respectively lower than 2 and 10%, while at the concentration level of 10 µg/mL (lower reporting limit) the RSD% was about 15% (Table S2). Regarding Nile Red assay, the mean coefficient of determination of the calibration curve computed in the range from 0 to 20 µg/mL of PC standards in water on seven model calibration curves after almost one year of application of the method was 0.985 ± 0.015. The curve equation, represented by the mean parameters ± confidence interval at 95% probability, resulted in y = (3769 ± 62) × (3567 ± 664) (Table S3). The analysis of the residuals showed that the distribution of the residuals was in all cases normal, thus evidencing that no systematic error affected the calibrations (data not shown). Method accuracy was demonstrated measuring samples at low, medium and high levels of concentration, repeating the determinations three times for each level of concentration on independent samples at 1, 10, and 20 µg/mL. The recovery percentages were always between 95 and 105% (Table S4). The method repeatability was assessed during one session of work measuring 6 independent samples at the level of concentration considered as more representative for the samples, i.e., at 10 µg/mL. The relative standard deviation percentage (RSD%) was lower than 5% for the series of measurements and, as such, it was considered a satisfactory value for the intra-assay precision. The next day, the samples were re-analyzed to assess the intermediate precision (Table S5). The statistical analysis of the data showed that there are no differences between the mean values found for the concentration of the target analyte over the two days of measurements. The data collected also showed that on the second day of measurements the RSD% did not exceed the 5% level. In addition, considering the overall performance of the method as evidenced by the 12 measurements carried out after two days of study, it could be assessed that the overall precision was within the same limit (5%), and that no statistically significant differences were found between the mean values of the results after day 1 and after day 2 of the study.

**Figure 2:**
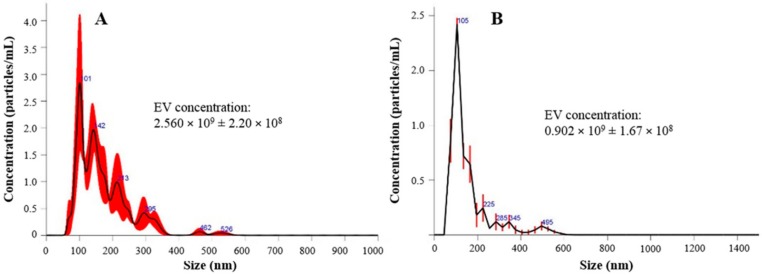
EVs particle concentration and size distribution before (**A**) and after (**B**) the lyophilization process. In red is indicated the ± standard error of the mean values (*n* = 3). EV concentration is expressed as mean value ± standard deviation (*n* = 3).

Particle size characterization revealed the presence of a heterogeneous EV population into both fresh and lyophilized products, which includes both exosomes (with diameter range reported in literature ranging between 40–120 nm) and microvesicles (250–1000 nm). In detail, the results revealed that the exosomes are not the most representative EV population. In fact, only 10% of the counted EVs have a diameter of less than 82.8 ± 2.0 nm, while 50% have a diameter of less than 172.8 ± 6.6 nm (after lyophilization) (Table 3 and Figure 2). As NTA technology is able to count the nanoparticles detected in a specific volume, we also measured the EV concentration before and after the lyophilization process; the particle concentration was slightly reduced after freeze-drying, probably because some EVs break up.

**Figure 3:**
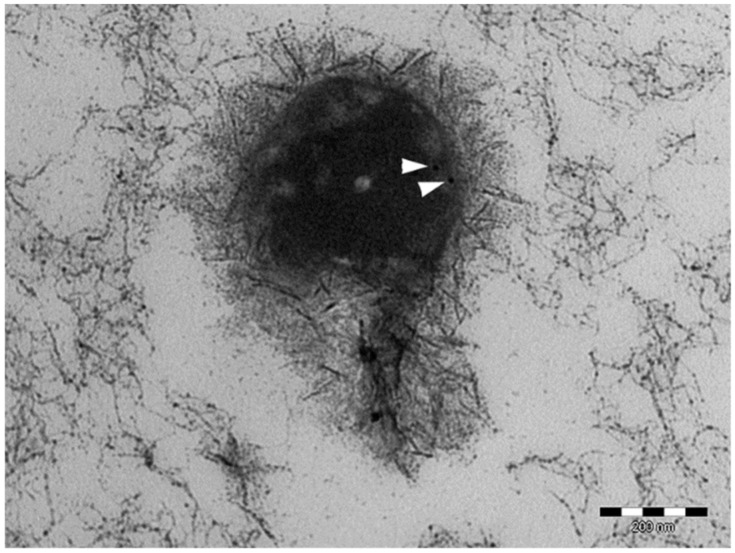
TEM image of ultrafiltered EV. The white arrows indicate the HSP90-reactive gold particles.

Morphological investigation showed that EV integrity and morphology were not altered by the ultrafiltration and lyophilization processes for all the produced batches. In particular, the preservation of EV integrity at the end of the ultrafiltration process was proven by TEM images, where EVs appeared to maintain their whole phospholipidic bilayer. In TEM images, ultrafiltered EVs were also positive for the HSP90-reactive gold particles (Figure 3).

**Figure 4:**
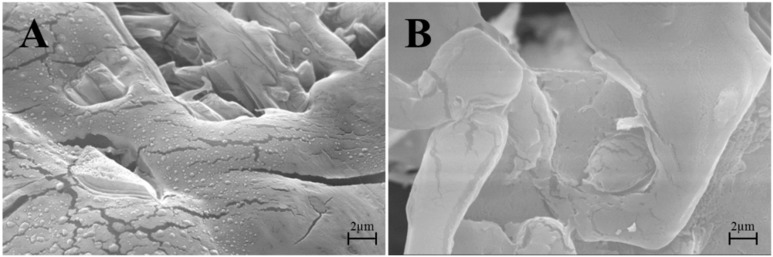
(**A**) SEM images of a lyo-secretome batch or (**B**) ultrafiltered and freeze-dried unconditioned media.

SEM images in Figure 4A show that at the end of the freeze-drying process, EVs maintained their integrity and spherical structure with a smooth surface; in Figure 4B ultrafiltered and freeze-dried conditioned media were reported as a negative control, indicating the absence of nanovesicular structures.

**Figure 5:**
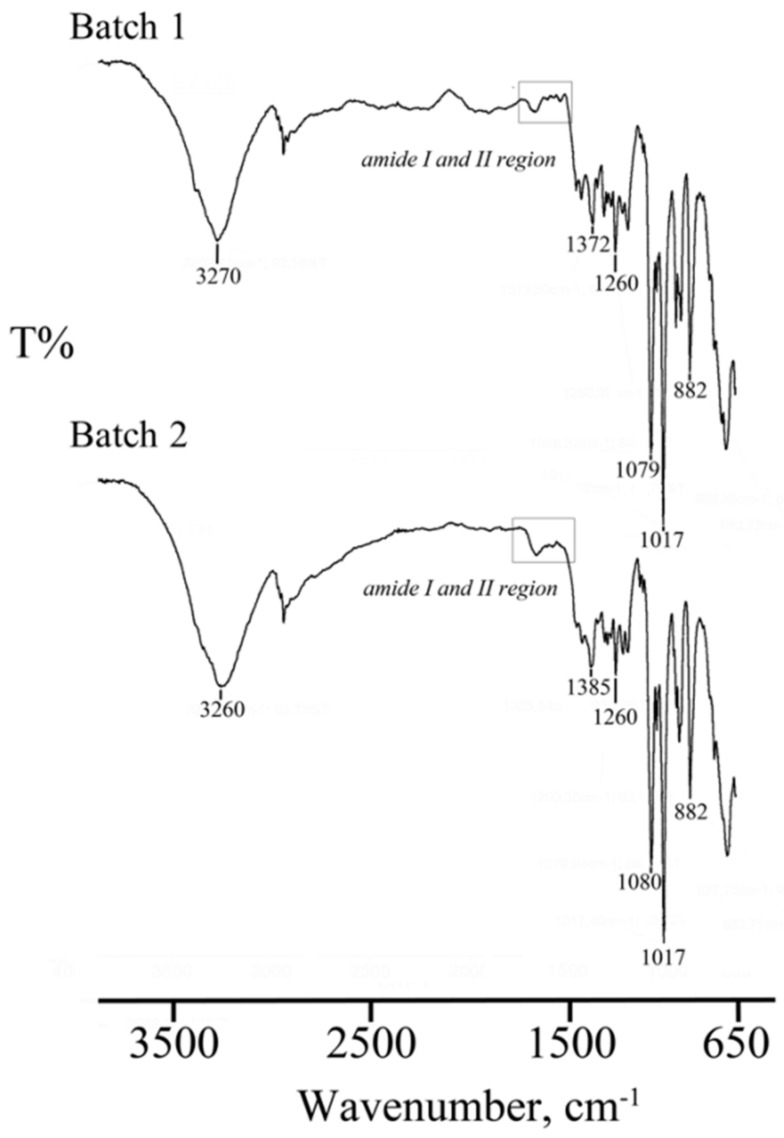
FTIR spectra of two lyo-secretome batches (batch 1 and batch 2). In the picture are evidenced the regions corresponding to Amide I and Amide II stretching vibrations at 1653 cm^−1^ and 1547 cm^−1^, respectively, confirming the presence of EVs and proteins in the lyophilized products.

FTIR spectra of two lyo-secretome batches are shown in Figure 5. The low-intensity bands around 1653 cm^−1^ and 1547 cm^−1^ due to the presence of amide I C=O stretching vibrations and amide II N-H banding vibrations of the peptide groups, respectively, confirmed the presence of EVs in the lyophilized products. Absorbance bands at around 1457 cm^−1^ and 1377 cm^−1^ related to CH_2_ and CH_3_ groups, confirmed the presence of lipid and protein. The bands detected in the spectral region between 1260 and 880 cm^−1^ are typical of phospholipids, triglycerides and cholesterol esters stretching vibrations, but unfortunately, this spectral region is superimposed to vibrations bands of the cryoprotectant used.

**Figure 6:**
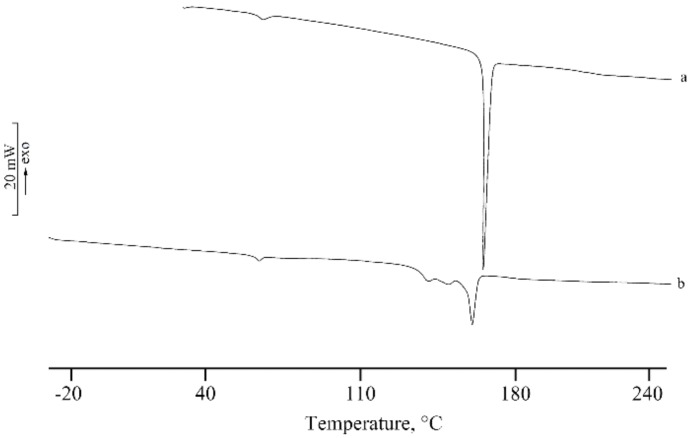
DSC of the phase of lyophilized commercial mannitol (curve a) and lyo-secretome (curve b).

DSC thermal profile of the solid lyo-secretome, reported in Figure 6 (curve b), showed a first endothermic peak at 67.6 ± 0.9 °C due to a desolvation effect followed by an endothermic effect at 161.5 ± 0.5 °C attributable to the melting of the anhydrous mannitol that resulted as stability in the β form, the same solid phase of the commercial product lyophilized (curve a); the broadening of the effect is probably due to the presence of the other components in the lyo-secretome product that resulted as impurity. The TGA curve showed a mass loss of 1.8 ± 0.2% in the temperature range between 50 and 80 °C, in correspondence to the first endothermic effect in the DSC profile. This effect is due to dehydration (curve not reported). Therefore, it is possible to assume the formation of a hydrate mannitol during the freeze-drying (FD) process, although the mass loss is lower than the theoretical loss of hemihydrate (4.71%), reported in the literature as mannitol solvatomorph [40,41]. The simulation of the freeze-drying process with DSC revealed the presence of the crystallization and consequent endothermic melting of freezing water without any thermal effects due to eutectic eventually formed between components (curve not reported).

**Figure 7:**
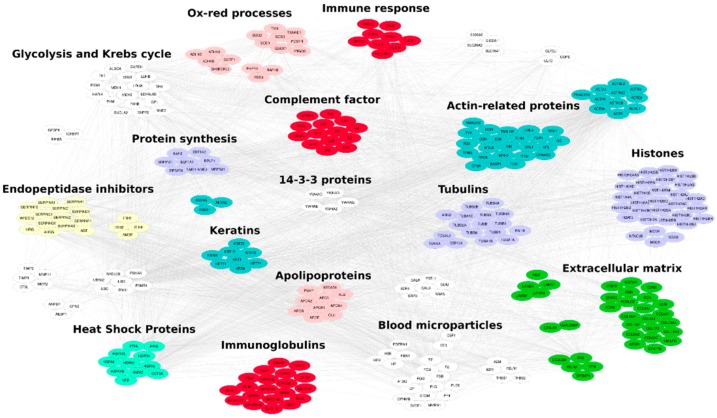
Protein-protein interaction (PPI) network reconstructed by considering the proteins identified by analyzing the lyo-secretome obtained by adipose-derived mesenchymal stem cells. The network counts 314 proteins and 3836 interactions retrieved from the major PPI repositories. Functional modules were selected by using Bingo 2.44 Cytoscape plugin. Red nodes: Immune system, Pink nodes: Oxidation-reduction processes, Violet nodes: Genetic Information Processing, Green nodes: Extracellular matrix, Light green nodes: Heat Shock Proteins, Yellow nodes: Endopeptidase inhibitors, Blue nodes: Cytoskeleton, White nodes: Others.

A preliminary characterization of the protein content allowed the identification of 349 distinct proteins (Table S6). The sample was analyzed in twice technical replicates for a total of six LC-MS/MS runs. All replicates showed very good repeatability with R^2^ values > 0.99 (Figure S2A–C) and most proteins (56%) were identified in all analyzed samples, while the remaining showed a frequency of identification <100% (Figure S2D and Table S6). About 57% of proteins were identified with an average SpC > 1 (Table S6, Figure S2E,F). The average SpC of each identified protein was normalized based on molecular weight (MW), to estimate a ranking of the most abundant proteins present in the analyzed samples (Table S6, Figure 7).

The characterized protein profile was functionally evaluated and the most enriched cellular components, molecular functions and biological processes were extracted (Figure S3). Of note, most proteins were vesicle/exosome and extracellular matrix annotated. At the functional level they were involved in immune response, response to stress, cytoskeleton and metabolism. In fact, the protein families most represented were structural proteins, heat shock proteins (HSPs), and endopeptidase inhibitors, as well as proteins involved in oxidative stress response (Figure S2, Figure 7).

### 3.3. Lyo-Secretome Safety

**Figure 8:**
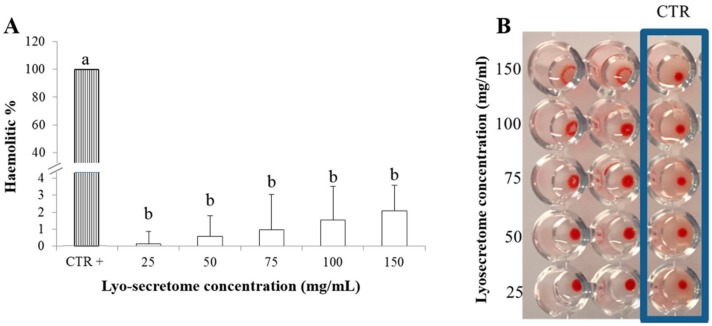
(**A**) In vitro lyo-secretome hemolytic test, mean values ± standard deviation (*n* = 3). Different letters indicate statistical differences between the means (*p* < 0.05). (**B**) In vitro hemagglutination assay.

With regard to safety, lyo-secretome was not hematolytic at any of the tested concentrations. The higher hemolytic percentage value of 2.11 ± 1.473 (mean value ± standard deviation, *n* = 3) was recorded for the highest concentration considered (150 mg/mL). This is also the only concentration tested that induced blood hemagglutination (Figure 8). Blood hemagglutination occurs when sialic acid receptors exposed on the surface of RBCs bind to the hemagglutinin glycoprotein which are generally found on the surface of the influenza virus, nanoparticles or EVs. After this binding, an agglutinated lattice is formed and it maintains the RBCs in a suspended distribution, typically viewed as a diffuse reddish solution. The formation of ‘button like’ structures, instead, confirms EV compatibility with hemoglobin because no lattice structure is formed.

**Figure 9:**
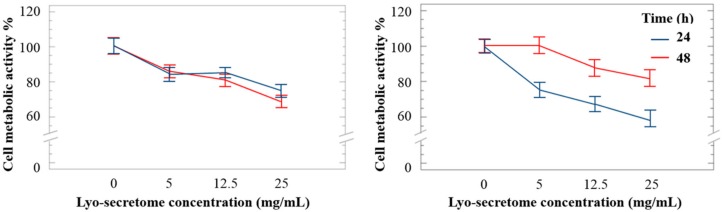
(**A**) Cell metabolic activity of human fibroblasts and (**B**) human chondrocytes treated with different doses of lyo-secretome after 24 (blue line) and 48 (red line) h. Mean values ± LSD (*n* = 3), ANOVA.

**Figure 10:**
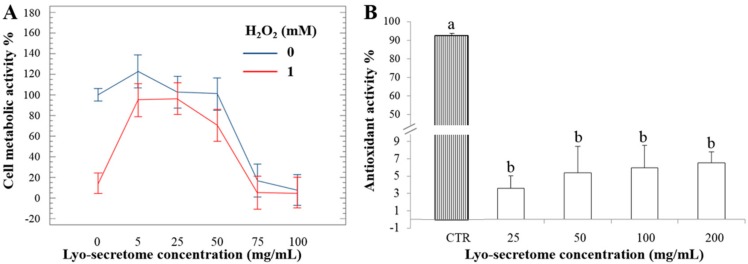
(**A**) Cell metabolic activity of NP cells treated with lyo-secretome with (red line) and without (blue line) H_2_O_2_. Mean values ± LSD (*n* = 3), ANOVA. (**B**) Lyo-secretome antioxidant activity % measured in terms of radical scavenging by DPPH assay, mean values ± standard deviation (*n* = 3). Different letters indicate statistical differences between the means (*p* < 0.05).

Lyo-secretome cytocompatibility was tested considering three different cell lines: HF, HC and NP cells. A dose-dependent reduction in cell metabolic activity was observed for all cell lines. However, cell metabolic activity remained ≥60% also at the highest concentration tested. Surprisingly, the cell metabolic activity of HC was higher after 48 h of incubation rather than after 24 h, suggesting that, for this cell line, exposition to lyo-secretome stimulated cell proliferation and, thus, metabolic activity (Figure 9). For NP cells, lyo-secretome become cytotoxic at a concentration of over 50 mg/mL (Figure 10A, blue line).

### 3.4. Lyo-Secretome Potency

By DPPH assay, lyo-secretome did not show ROS-scavenging activity, even at the highest concentration tested. In fact, the antioxidant activity % was less than 10% compared to ascorbic acid considered as the positive control (Figure 10B). No statistical differences were found between the concentrations tested (*p* > 0.05).

Next, the ability of lyo-secretome to protect cells from the damage induced by oxidative stress stimulation was evaluated. Oxidative stress results on NP cells showed that after H_2_O_2_ addition, cell metabolic activity decreased, confirming the suitability of the adopted experimental conditions. At lower doses (5, 25 and 50 mg/mL) lyo-secretome protects cells from oxidative stress damage (Figure 9B, red line), but at higher doses (75 and 100 mg/mL) the cell metabolic activity is dramatically reduced (Figure 10B, blue line), probably due to a cytotoxic effect. Therefore, for doses between 5 and 50 mg/mL, lyo-secretome protected NP cells from the oxidative stress damages induced by H_2_O_2_.

## 4. Discussion

In this work, we propose a method to transform MSC-secretome into a pharmaceutical product, for its large-scale production. For this purpose, ultrafiltration and freeze-drying were combined. Ultrafiltration was chosen because, compared to chromatographic methods, it is characterized by lower costs, shorter times and easier scalability for industrial production [42]. Moreover, it allows the choice of filtration modules with a different MWCO and thus retention of only EVs or the whole secretome. In detail, we chose a MWCO sufficiently low to retain both EVs and soluble proteins. Lyophilization is used to guarantee the long-term stability and easy storage and reconstitution of products. This process generates a variety of freezing and drying stresses that can alter the stability of the biological samples. Therefore, during freeze-drying, a cryoprotectant is needed to protect the protein and EV lipidic layer from the damages produced by ice crystals during the freezing step and to inhibit the vesicle fusion/aggregation following dehydration. For the lyo-secretome formulation, we chose mannitol as a cryoprotectant. It belongs to the sugar class, and its precise mechanisms in stabilizing biological membrane during lyophilization remain a matter of debate, although two hypotheses have been proposed [43]. Advantageously, the selected cryoprotectant was able to partially maintain EV integrity, as revealed by morphological and ultrastructural investigations, and to prevent aggregation, as revealed by NTA analysis performed before and after the lyophilization. EV integrity is also favored by the lower stresses generated during the ultrafiltration process, compared to the high shear forces of ultracentrifugation (which is largely employed for the EV isolation). Likewise, Nordin et al. have proved that ultrafiltration produced significantly higher EV yield compared to ultracentrifugation, and, more importantly, vesicle structure was not affected [44]. Overall, the maintenance of the whole phospholipidic layer is an important aspect, as it provides a protected and controlled internal microenvironment, allowing cargo (including the easily-degradable genetic material) to travel long distances within tissues without degradation [45].

In the lyophilized product, exosomes were less abundant than microvesicles. In our opinion, this can be related to two different reasons: (i) it is probable that, during the ultrafiltration process, exosomes are more adsorbed on the membrane of filtration module, due to their reduced dimension; and, (ii) for exosomes, the forces generated during the ultrafiltration and freeze-drying processes are distributed on a reduced superficial area, and thus they break up more easily than microvesicles.

It is well known that large amounts of EVs are normally present in FBS [46]. Therefore, great attention must be paid to assure that no contaminants are introduced by the use of culture media and supplements during the cell expansion phase. Based on the evidence that Gronthos et al. found the presence of the HSP90 on the surface of the MSC membrane [28], the TEM image confirmed that EVs in lyo-secretome are derived from MSCs.

A physical-chemical characterization was performed to define the pharmaceutical quality of lyo-secretome. ATR-FTIR spectroscopy was recently reported by Mihaly et al. to investigate different subpopulations of EVs [47]. By means of this technique, we revealed the simultaneous presence of proteins and lipids. Overall, FTIR spectra of all samples were overlapping, thus generating a sort of fingerprint in terms of protein and lipid composition, confirming batch-to-batch reproducibility. The DSC thermal profile of the solid lyo-secretome showed a first endothermic effect due to desolvation, as suggested also by the TGA mass loss, followed by a broad endothermic effect attributable to the melting of the anhydrous mannitol. The lyophilization process successfully occurred without interaction between the formulation components, as suggested by the absence of any thermal effect related to eutectic melting or glass transition events.

The few clinical trials performed using the paracrine factors of MSCs seem to have already established the safety and feasibility of MSC-secretome, as none reported related adverse effects. Moreover, the administration of MSC-EVs to different animal models did not cause any adverse reaction [20]. However, as MSC-secretome is considered a pharmaceutical product, subject to technological processes and added with a cryoprotectant, in vitro safety evaluation is mandatory, and must also considering the means of administration. The intravenous injection (i.v.) is the most commonly used means as it allows EVs to reach their target anywhere in the body. However, the short half-life index of EVs after injection is one of the major limitations; several studies have demonstrated that the detectable EVs decrease by 50% in a time range between 2 and 30 min after administration [45,48]. Therefore, strategies to improve their plasma half-life are needed; the most promising seems to be the surface modification of EVs with targeting antibodies [44]. As required for i.v. administration, in this study we demonstrated that lyo-secretome does not induce hemolysis until reaching a concentration of 150 mg/mL, and thus can be considered hemocompatible according to ISO/TR 7406 [48]. Cytocompatibility of the product was demonstrated for HF and HC (up to 25 mg/mL) and for NP cells (up to 50 mg/mL) according to ISO 10993-5. In current medicinal product legislation frameworks, secretome and EVs remain in a regulatory gap; they are not cells, but cellular products, thus they cannot be considered as Advanced Therapy Medicinal Products as their parental cells. Although the US Food and Drug Administration or European Medicines Agency have yet to regulate secretome-based products, according to a position paper of the International Society of Extracellular Vesicles (ISEV), the studied products could probably be considered as biological or biotechnological medicinal products [21].

It has been indicated by experimental evidence that oxidative stress is linked to inflammation and inflammatory responses and plays an important role in the pathophysiology of different diseases [49]. Although, lyo-secretome showed no free-radical scavenging activity, as revealed by DPPH assay, it was able to protect cells from the damage induced by oxidative stress in a concentration range between 5 and 50 mg/mL. This ability is probably related to the induction and regeneration of the antioxidant systems that are physiologically present in cells. In this regard, both MSCs and their EVs were previously investigated and showed antioxidant effects. Kim et al., studying MSCs as an alternative to glucocorticoid steroids for the treatment of spinal cord injury, found that damage was reduced through a cell-mediated enhancement of anti-oxidative and anti-inflammatory mechanisms [50]. Zhang G et al. tested the anti-oxidation effect of MSC-EVs as an effective strategy for curing acute kidney injury (AKI) and found decreased oxidative stress mediated by an enhancement of Nrf2/antioxidant response element and HO-1 up-regulation [51]. Finally, Nightingale et al. observed that among the soluble factors secreted by MSCs there was also SOD3, which is an antioxidant enzyme that dismutes the toxic free radicals produced during the inflammatory processes [52]. All these observations are in agreement with the proteomic content found in our samples. Among the most represented protein categories, we found proteins involved in immune and stress response, such as SOD1, SOD2 and SOD3 involved in oxidative stress response. In fact, MSCs exert, in vivo, a pivotal role in maintaining tissue homeostasis. In the proximity of a damaged tissue, and thus exposed to lower levels of oxygen and nutrients, MSCs react by producing a pool of bioactive compounds to start the repairing and regeneration processes. In vitro, such condition is reproduced by serum starvation [41] or hypoxia [42]. It is reasonable that the secretome produced by MSCs by serum starvation is different, in terms of molecular composition, from secretome produced by hypoxic conditions. As an example, Riis et al. found that a relatively small fraction of the proteins produced by MSCs was significantly affected by hypoxia, and especially the proteins involved in extracellular matrix synthesis and cell metabolism [43]. More evidence from the literature shows that hypoxia-preconditioning enhances the angiogenic capacity of secretome, in addition to the regenerative and immunomodulatory abilities [47,48].

Finally, it must also to be considered that the composition of MSC-secretome can be modulated, according to the therapeutic purpose, by preconditioning the MSCs during in vitro culture [53,54]. For example, extensive evidence now exists to support how the exposition of MSCs to pro-inflammatory stimuli can enhance the immunomodulant and regenerative potential of their secretome [55,56,57,58,59,60]. It is our opinion that, in the very near future, the MSCs will be “educated” to produce the best secretome according to the desired therapeutic goals.

## 5. Conclusions

Secretome-based therapeutics represent a promising approach for treating several diseases. Although MSC-secretome efficacy has been widely established in numerous preclinical models, the development of large-scale GMP-grade secretome-based pharmaceuticals, required for future clinical trials, is still missing. With this work, we propose a GMP-compliant integrated process of ultrafiltration and lyophilization able to isolate and freeze-dry MSC-secretome. The successful isolation and lyophilization processes allow the production of ready-to-use product without alteration of the EV morphology and integrity. Due to the easy scale-up, this process can even be performed on larger amounts of supernatants enriched in secretome. This allows the production of lyo-secretome from larger amounts of cells, thus reducing variability in terms of protein and lipid content and allowing for a more standardized product. Lyo-secretome was also shown to be safe and effective against the damage induced by oxidative stress. This efficacy is also supported by the proteomic characterization we performed: proteins involved in immune and oxidative stress response were among the most represented. The product can be also employed as a new bio-inspired natural carrier for drug delivery, exploiting the homing properties of EVs. In fact, as the process was revealed to maintain the integrity of the EV lipid layer, easy-degradable drugs can be encapsulated, being protected in an internal and controlled microenvironment by EVs. Finally, a highly pure, “ready-off-the-shelf”, well-characterized and stable powder-based EV standard could be the missing piece necessary for scientific progress in this field and for group-to-group comparisons.

## 6. Patents

Perteghella, S., Bari, E., Chlapanidas, T., Sorlini, M., De Girolamo, L., Perucca Orfei, C., Viganò, M. and Torre, M.L. Process for isolating and freeze-drying extracellular vesicles. WO2018078524 (A1).

## Figures and Tables

**Table 1:** Lyo-secretome batches report.

Batch n.	Cell Source Type	Cell Line	Total Cell Number	Cell Viability %
1	ASCs	AM16	85 × 10^6^	98.2
2	ASCs	AM21	82 × 10^6^	96.6
3	ASCs	AM18	121 × 10^6^	98.0

**Table 2:** Lyo-secretome total protein and lipid content; mean values ± standard deviation, *n* = 3. Different letters indicate a significant difference between groups (*p* < 0.0001).

Batch n.	Yield (mg)	Cell Equivalent/mg (×10^6^)	µg Protein/mg lyo-Secretome	µg Lipid/mg lyo-Secretome
1	882	0.096	59.7 ± 2.053 ^a^	8.9 ± 0.137 ^a^
2	828	0.098	69.2 ± 1.332 ^b^	4.9 ± 0.304 ^b^
3	1115	0.104	21.7 ± 0.595 ^c^	1.9 ± 0.885 ^c^

**Table 3:** EV particle size distribution and concentration before and after the lyophilization process (mean values ± standard deviation, *n* = 3). The EV particle concentration per mL corresponds to the EV particle concentration per mg, as for analysis freeze-dried samples were re-suspended to a final concentration of 1 mg/mL.

Batch	Mean Diameter	Mode (nm)	d_10_ (nm)	d_50_ (nm)	d_90_ (nm)	Particle Concentration Per mL/mg of Product
Before lyophilization	171.8 ± 18.3	138.1 ± 38.8	97.7 ± 9.8	158.8 ± 21.1	282.1 ± 22.6	2.560 × 10^9^ ± 2.20 × 10^8^
After lyophilization	231.1 ± 10.2	152.5 ± 15.2	82.8 ± 2.0	172.8 ± 6.6	358.4 ± 33.6	0.902 × 10^9^ ± 1.67 × 10^8^

## Supplementary Materials

Supplementary Materials are available online at http://www.mdpi.com/2073-4409/7/11/190/s1.
