# Supplementary material for: Pilot Production of Mesenchymal Stem/Stromal Freeze-Dried Secretome for Cell-Free Regenerative Nanomedicine: A Validated GMP-Compliant Process
Source: Cells. 2018 Oct 30;7(11):190. doi: 10.3390/cells7110190 (PMC6262564; doi:10.3390/cells7110190)
Supplement: Supplementary file 1 [file cells-07-00190-s001.pdf]

Supplementary materials:
Pilot production of mesenchymal stem/stromal freeze-dried secretome for cell-free regenerative nanomedicine: a validated GMP-compliant process

Elia Bari, Sara Perteghella, Dario Di Silvestre, Marzio Sorlini, Laura Catenacci, Milena Sorrenti, Giorgio Marrubini, Rossana Rossi, Giuseppe Tripodo, Pierluigi Mauri, Mario Marazzi and Maria Luisa Torre

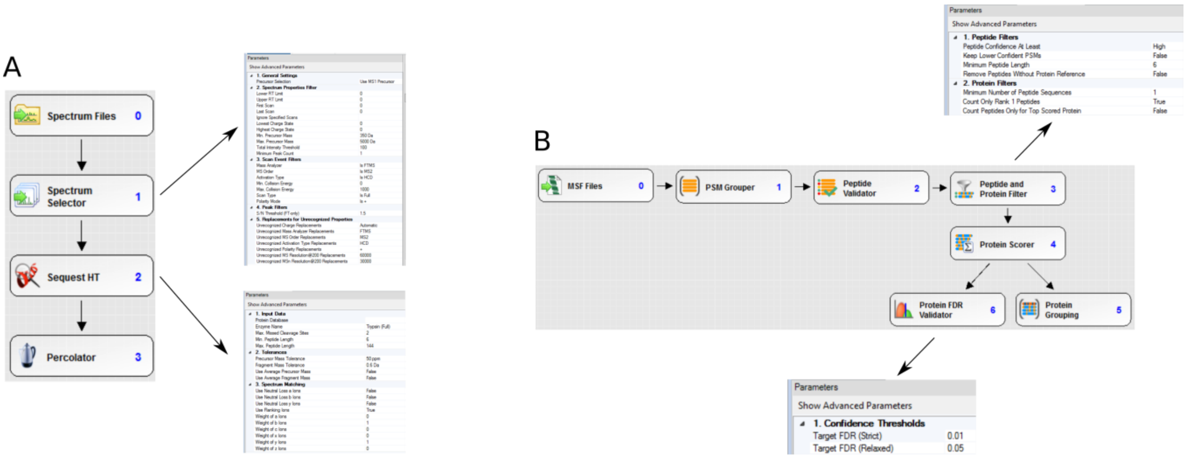

Figure S1. Experimental MS/MS data processing workflows based on Discoverer 2.1 software. A) Processing workflow and parameters set for processing MS/MS spectra. B) Consensus workflow and parameters set for filtering the most confident proteins and peptides.

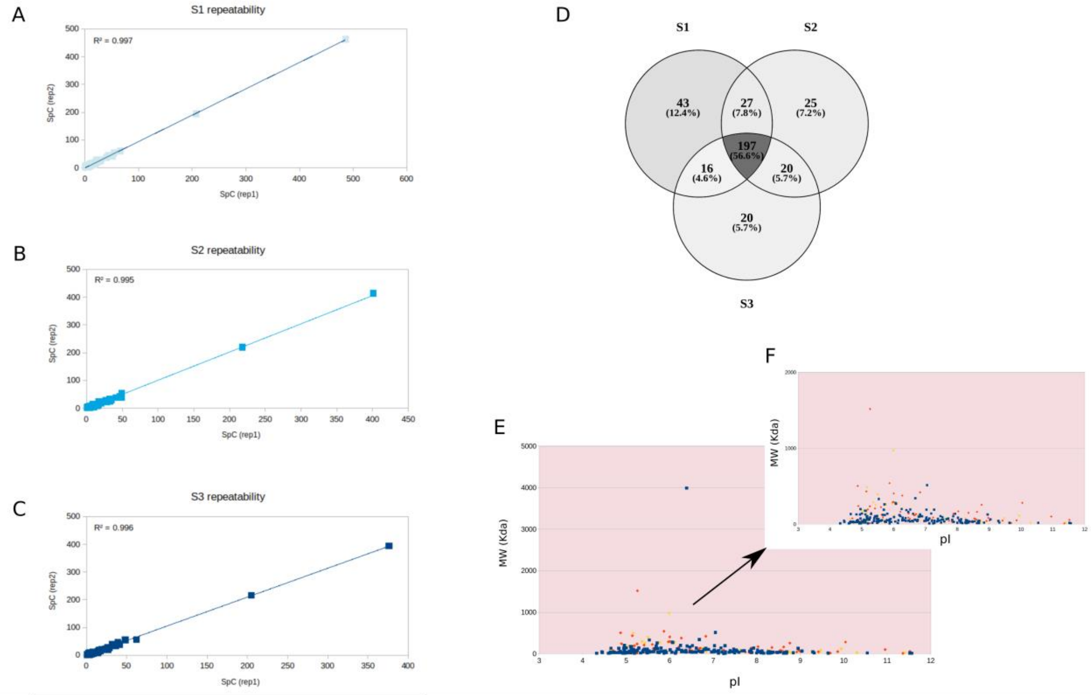

Figure S2. Protein profile of the lyo-secretome obtained by adipose-derived mesenchymal stem cells. Repeatibility of S1 (A), S2 (B) and S3 (C) technical replicate analyses; in all cases R2>0.99. D) Venn diagram of proteins identified in S1, S2 and S3 biological replicates. E, F) Virtual 2D map obtained plotting MW and pI of the proteins identified; blue dots: proteins identified with an average SpC<1, red dots: proteins identified with an average 2<SpC<10, yellow dots: proteins identified with an average SpC>10.

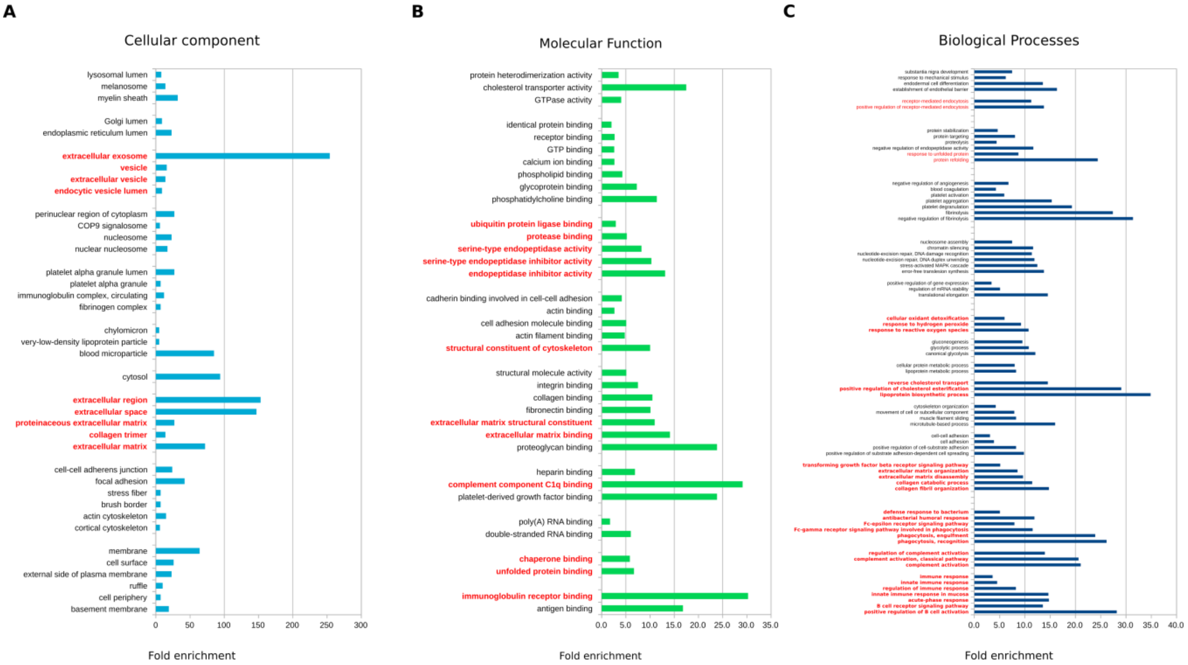

Figure S3: A) Cellular components, B) molecular functions and C) biological processes enriched in the proteome characterized by analyzing the lyo-secretome obtained by adipose-derived mesenchymal stem cells. Fold enrichment was calculated by means of DAVID db as reported in M&M section. In red are marked the most enriched categories.

Table S1. Model calibration curves for BCA-protein assay.

Date	Slope	Intercept	R^2	Range (µg/ml)	LOD (µg/ml)	LOQ (µg/ml)	Accuracy	
							Min	Max
17.5	0,0171	0,65	0,975	20-100	20	60	93	112
18.5	0,0170	0,13	0,998	2.5-30	1,4	4	97	108
19.5	0,0248	0,57	0,998	20-50	2,3	7	96	102
23.5	0,0129	0,16	0,974	2.5-50	6	18	89	105
24.5	0,0109	0,24	0,951	10-50	14	44	92	113
24.5	0,0135	0,26	0,954	10-50	14	42	92	113
1.6	0,0098	0,18	0,993	10-50	5	16	98	104
15.6	0,0159	0,15	0,998	2.5-50	1,6	5	96	102
28.6	0,0123	0,41	0,993	5-50	4	13	95	103

Table S2. Intermediate precision for BCA-protein assay at high, intermediate and low level.

**High level**			**Intermediate level**			**Low level**		
	Day 1	Day 2		Day 1	Day 2		Day 1	Day 2
**50 µg/ml**	0,669	0,653	**30 µg/ml**	0,481	0,426	**10 µg/ml**	0,260	0,205
	0,683	0,650		0,467	0,437		0,271	0,202
	0,669	0,674		0,493	0,456		0,277	0,204

**Variance analysis: one factor**			**High level: 50 µg/ml**				
			Summary				
Groups	*Count*	*Sum*	*Mean*	*Variance*			Ok at 95%
Day 1	3	2,021	0,673667	6,53E-05			
Day 2	3	1,977	0,659	0,000171			
Origin of the variation	*SQ*	*gdl*	*MQ*	*F*	*Value of significance*	*F crit*	*Prob.*
Between groups	0,000323	1	0,000323	2,730606	0,173786	7,708647	95%
In groups	0,000473	4	0,000118				
Total	0,000795	5					

**Variance analysis: one factor**			**Intermediate level: 30 µg/ml**				
			Summary				
Groups	*Count*	*Groups*	*Count*				Ok at 99%
Day 1	3	1,441	0,480333				
Day 2	3	1,319	0,439667				
Origin of the variation	*SQ*	*gdl*	*MQ*	*F*	*Value of significance*	*F crit*	*Prob.*
Between groups	0,002481	1	0,002481	12,41368	0,024377	7,708647	95%
In groups	0,000799	4	0,0002			21,19769	99%
Total	0,00328	5					

**Variance analysis: one factor**			**Low level: 10 µg/ml**				
			Summary				
Groups	*Count*	*Sum*	*Mean*	*Variance*			No
Day 1	3	0,808	0,269333	7,43E-05			
Day 2	3	0,611	0,203667	2,33E-06			
Origin of the variation	*SQ*	*gdl*	*MQ*	*F*	*Value of significance*	*F crit*	*Prob.*
Between groups	0,006468	1	0,006468	168,7348	0,000203	21,19769	99%
In groups	0,000153	4	3,83E-05			74,13729	99,9%
Total	0,006622	5					

Table S3. Linearity model calibration curves for Nile Red assay.

Phosphatidylcholine concentration (μg/ml)	Fluorescence reading
20	78832
15	59336
10	41027
8	35988
2.5	12250

Table S4. Accuracy data for Nile Red assay.

Concentration level (µg/ml)	Concentration measured (µg/ml)	Recovery
1.00	0.97	96.7
	0.98	97.8
	0.98	98.4
10.00	9.85	98.5
	9.98	99.8
	10.1	101.1
20.00	20.1	100.5
	19.9	99.5
	20.6	102.8

Table S5. Precision data for Nile Red assay.

	Concentration found (µg/ml)	
	0.97	96.7
	0.98	97.8
	0.98	98.4
	9.85	98.5
	9.98	99.8
	10.1	101.1
	20.1	100.5
Mean	10.2	10.0
s	0.4	0.3
Confidence interval (95%, n=6)	0.46	0.36
RSD %	4.28	3.45
Total mean	10.1	
Pooled s	0.4	
Confidence interval (95%, n=12)	0.27	
RSD %	4.15	
Fcalc(0.05;5;5)	1.592	
F(0.05; 5;5)	5.050	
tcalc(0.05; 10)	0.820	
tcrit(0.05; 10)	2.228	

Table legend

Table S6. List of proteins identified by analyzing the lyo-secretome obtained by adipose-derived mesenchymal stem cells. S1, S2, S3: biological replicate samples. SpC: spectral count. MW: molecular weight, Frequency of identification (%). n=6.

Annotations			Spectral count (SpC)									Normalized SpC
Accession	Description	Gene Name	S1_1	S1_2	S2_1	S2_2	S3_1	S3_2	Frequency of Identification (%)	Average SpC	MW	(Av.SpC/MW)*1000
P02768	Serum albumin OS=Homo sapiens GN=ALB PE=1 SV=2	ALB	486	462	401	414	376	394	100,0	422,17	69,367	6,09E+00
P02751	Fibronectin OS=Homo sapiens GN=FN1 PE=1 SV=4	FN1	207	194	218	220	205	216	100,0	210,00	262,625	8,00E-01
P01024	Complement C3 OS=Homo sapiens GN=C3 PE=1 SV=2	C3	66	61	49	53	62	56	100,0	57,83	187,148	3,09E-01
P01023	Alpha-2-macroglobulin OS=Homo sapiens GN=A2M PE=1 SV=3	A2M	51	41	47	42	48	55	100,0	47,33	163,291	2,90E-01
P02787	Serotransferrin OS=Homo sapiens GN=TF PE=1 SV=3	TF	54	54	42	38	41	39	100,0	44,67	77,064	5,80E-01
P12109	Collagen alpha-1(VI) chain OS=Homo sapiens GN=COL6A1 PE=1 SV=3	COL6A1	41	39	41	38	36	34	100,0	38,17	108,529	3,52E-01
P01009	Alpha-1-antitrypsin OS=Homo sapiens GN=SERPINA1 PE=1 SV=3	SERPINA1	40	37	32	34	39	46	100,0	38,00	46,737	8,13E-01
P02647	Apolipoprotein A-I OS=Homo sapiens GN=APOA1 PE=1 SV=1	APOA1	43	44	35	29	28	27	100,0	34,33	30,778	1,12E+00
P08123	Collagen alpha-2(I) chain OS=Homo sapiens GN=COL1A2 PE=1 SV=7	COL1A2	28	23	32	29	32	40	100,0	30,67	129,314	2,37E-01
P04114	Apolipoprotein B-100 OS=Homo sapiens GN=APOB PE=1 SV=2	APOB	20	21	49	40	26	28	100,0	30,67	515,605	5,95E-02
P60709	Actin, cytoplasmic 1 OS=Homo sapiens GN=ACTB PE=1 SV=1	ACTB	22	28	33	25	28	27	100,0	27,17	41,737	6,51E-01
P63261	Actin, cytoplasmic 2 OS=Homo sapiens GN=ACTG1 PE=1 SV=1	ACTG1	22	28	33	25	28	27	100,0	27,17	41,793	6,50E-01
P01857	Ig gamma-1 chain C region OS=Homo sapiens GN=IGHG1 PE=1 SV=1	IGHG1	22	26	27	28	28	27	100,0	26,33	36,106	7,29E-01
P08670	Vimentin OS=Homo sapiens GN=VIM PE=1 SV=4	VIM	30	28	28	24	24	24	100,0	26,33	53,652	4,91E-01
P02675	Fibrinogen beta chain OS=Homo sapiens GN=FGB PE=1 SV=2	FGB	28	28	23	24	26	27	100,0	26,00	55,928	4,65E-01
P08253	72 kDa type IV collagenase OS=Homo sapiens GN=MMP2 PE=1 SV=2	MMP2	25	24	26	24	26	27	100,0	25,33	73,882	3,43E-01
P12111	Collagen alpha-3(VI) chain OS=Homo sapiens GN=COL6A3 PE=1 SV=5	COL6A3	23	25	31	25	27	21	100,0	25,33	343,669	7,37E-02
C9JC84	Fibrinogen gamma chain OS=Homo sapiens GN=FGG PE=1 SV=1	FGG	24	28	21	22	19	22	100,0	22,67	52,338	4,33E-01
P36955	Pigment epithelium-derived factor OS=Homo sapiens GN=SERPINF1 PE=1 SV=4	SERPINF1	24	24	24	22	20	21	100,0	22,50	46,312	4,86E-01
P01834	Ig kappa chain C region OS=Homo sapiens GN=IGKC PE=1 SV=1	IGKC	23	20	20	18	20	19	100,0	20,00	11,765	1,70E+00
P02671	Fibrinogen alpha chain OS=Homo sapiens GN=FGA PE=1 SV=2	FGA	20	19	17	23	16	20	100,0	19,17	94,973	2,02E-01
P02452	Collagen alpha-1(I) chain OS=Homo sapiens GN=COL1A1 PE=1 SV=5	COL1A1	19	14	16	15	17	16	100,0	16,17	138,941	1,16E-01
P01871	Ig mu chain C region OS=Homo sapiens GN=IGHM PE=1 SV=3	IGHM	15	17	15	15	15	14	100,0	15,17	49,440	3,07E-01
P68032	Actin, alpha cardiac muscle 1 OS=Homo sapiens GN=ACTC1 PE=1 SV=1	ACTC1	12	15	17	14	15	16	100,0	14,83	42,019	3,53E-01
P68133	Actin, alpha skeletal muscle OS=Homo sapiens GN=ACTA1 PE=1 SV=1	ACTA1	12	15	17	14	15	16	100,0	14,83	42,051	3,53E-01
P51884	Lumican OS=Homo sapiens GN=LUM PE=1 SV=2	LUM	13	16	16	12	16	15	100,0	14,67	38,429	3,82E-01
D6RF35	Vitamin D-binding protein OS=Homo sapiens GN=GC PE=1 SV=1	GC	14	11	17	15	13	13	100,0	13,83	53,021	2,61E-01
Q15582	Transforming growth factor-beta-induced protein ig-h3 OS=Homo sapiens GN=TGFBI PE	TGFBI	18	15	9	12	15	12	100,0	13,50	74,681	1,81E-01
P0C0L5	Complement C4-B OS=Homo sapiens GN=C4B PE=1 SV=2	C4B	14	18	9	14	14	9	100,0	13,00	192,751	6,74E-02
P05155	Plasma protease C1 inhibitor OS=Homo sapiens GN=SERPING1 PE=1 SV=2	SERPING1	10	15	12	16	13	11	100,0	12,83	55,154	2,33E-01
P00738	Haptoglobin OS=Homo sapiens GN=HP PE=1 SV=1	HP	13	11	14	9	14	15	100,0	12,67	45,205	2,80E-01
P23142	Fibulin-1 OS=Homo sapiens GN=FBLN1 PE=1 SV=4	FBLN1	16	13	16	10	10	10	100,0	12,50	77,214	1,62E-01
P07585	Decorin OS=Homo sapiens GN=DCN PE=1 SV=1	DCN	11	10	11	12	14	13	100,0	11,83	39,747	2,98E-01
P19823	Inter-alpha-trypsin inhibitor heavy chain H2 OS=Homo sapiens GN=ITIH2 PE=1 SV=2	ITIH2	18	12	8	10	12	10	100,0	11,67	106,463	1,10E-01
Q9Y490	Talin-1 OS=Homo sapiens GN=TLN1 PE=1 SV=3	TLN1	10	10	13	13	13	10	100,0	11,50	269,767	4,26E-02
P09871	Complement C1s subcomponent OS=Homo sapiens GN=C1S PE=1 SV=1	C1S	11	10	10	12	13	12	100,0	11,33	76,684	1,48E-01
P63104	14-3-3 protein zeta/delta OS=Homo sapiens GN=YWHAZ PE=1 SV=1	YWHAZ	11	13	10	11	10	9	100,0	10,67	27,745	3,84E-01
A0A0G2JPR0	Complement C4-A OS=Homo sapiens GN=C4A PE=1 SV=1	C4A	0	18	9	14	14	9	83,3	10,67	192,876	5,53E-02
P00450	Ceruloplasmin OS=Homo sapiens GN=CP PE=1 SV=1	CP	11	8	11	12	8	12	100,0	10,33	122,205	8,46E-02
P02763	Alpha-1-acid glycoprotein 1 OS=Homo sapiens GN=ORM1 PE=1 SV=1	ORM1	9	10	9	8	12	11	100,0	9,83	23,512	4,18E-01
P06733	Alpha-enolase OS=Homo sapiens GN=ENO1 PE=1 SV=2	ENO1	8	11	10	11	10	9	100,0	9,83	47,169	2,08E-01
A0M8Q6	Ig lambda-7 chain C region OS=Homo sapiens GN=IGLC7 PE=4 SV=2	IGLC7	11	10	11	10	8	8	100,0	9,67	11,254	8,59E-01
P0CG06	Ig lambda-3 chain C regions OS=Homo sapiens GN=IGLC3 PE=1 SV=1	IGLC3	11	10	11	10	8	8	100,0	9,67	11,266	8,58E-01
P0CF74	Ig lambda-6 chain C region OS=Homo sapiens GN=IGLC6 PE=4 SV=1	IGLC6	11	10	11	10	8	8	100,0	9,67	11,277	8,57E-01
P0CG05	Ig lambda-2 chain C regions OS=Homo sapiens GN=IGLC2 PE=1 SV=1	IGLC2	11	10	11	10	8	8	100,0	9,67	11,294	8,56E-01
P04264	Keratin, type II cytoskeletal 1 OS=Homo sapiens GN=KRT1 PE=1 SV=6	KRT1	11	13	10	4	8	10	100,0	9,33	66,039	1,41E-01
B4DPQ0	Complement C1r subcomponent OS=Homo sapiens GN=C1R PE=1 SV=1	C1R	9	7	10	9	10	11	100,0	9,33	81,890	1,14E-01
P01861	Ig gamma-4 chain C region OS=Homo sapiens GN=IGHG4 PE=1 SV=1	IGHG4	10	9	12	0	12	12	83,3	9,17	35,941	2,55E-01
P12814	Alpha-actinin-1 OS=Homo sapiens GN=ACTN1 PE=1 SV=2	ACTN1	5	10	10	9	11	10	100,0	9,17	103,058	8,89E-02
P39059	Collagen alpha-1(XV) chain OS=Homo sapiens GN=COL15A1 PE=1 SV=2	COL15A1	4	9	13	10	6	9	100,0	8,50	141,720	6,00E-02
P01876	Ig alpha-1 chain C region OS=Homo sapiens GN=IGHA1 PE=1 SV=2	IGHA1	7	10	10	6	8	8	100,0	8,17	37,655	2,17E-01

P60174	Triosephosphate isomerase OS=Homo sapiens GN=TPI1 PE=1 SV=3	TPI1	7	9	6	9	9	8	100,0	8,00	30,791	2,60E-01
Q16363	Laminin subunit alpha-4 OS=Homo sapiens GN=LAMA4 PE=1 SV=4	LAMA4	11	9	9	8	3	7	100,0	7,83	202,524	3,87E-02
P26022	Pentraxin-related protein PTX3 OS=Homo sapiens GN=PTX3 PE=1 SV=3	PTX3	11	7	5	9	4	10	100,0	7,67	41,976	1,83E-01
P02790	Hemopexin OS=Homo sapiens GN=HPX PE=1 SV=2	HPX	8	7	7	9	5	9	100,0	7,50	51,676	1,45E-01
P01859	Ig gamma-2 chain C region OS=Homo sapiens GN=IGHG2 PE=1 SV=2	IGHG2	7	8	4	8	7	10	100,0	7,33	35,901	2,04E-01
P00338	L-lactate dehydrogenase A chain OS=Homo sapiens GN=LDHA PE=1 SV=2	LDHA	10	8	7	7	6	6	100,0	7,33	36,689	2,00E-01
P04217	Alpha-1B-glycoprotein OS=Homo sapiens GN=A1BG PE=1 SV=4	A1BG	9	9	5	9	6	6	100,0	7,33	54,254	1,35E-01
P21333	Filamin-A OS=Homo sapiens GN=FLNA PE=1 SV=4	FLNA	9	6	9	4	8	7	100,0	7,17	280,739	2,55E-02
G3XAI2	Laminin subunit beta-1 OS=Homo sapiens GN=LAMB1 PE=1 SV=1	LAMB1	8	7	8	6	7	5	100,0	6,83	200,478	3,41E-02
V9GYM3	Apolipoprotein A-II OS=Homo sapiens GN=APOA2 PE=1 SV=1	APOA2	6	8	5	8	7	6	100,0	6,67	14,914	4,47E-01
P67936	Tropomyosin alpha-4 chain OS=Homo sapiens GN=TPM4 PE=1 SV=3	TPM4	7	9	5	6	5	6	100,0	6,33	28,522	2,22E-01
Q12841	Follistatin-related protein 1 OS=Homo sapiens GN=FSTL1 PE=1 SV=1	FSTL1	5	6	7	7	7	6	100,0	6,33	34,986	1,81E-01
P10809	60 kDa heat shock protein, mitochondrial OS=Homo sapiens GN=HSPD1 PE=1 SV=2	HSPD1	6	3	8	6	5	8	100,0	6,00	61,055	9,83E-02
P05121	Plasminogen activator inhibitor 1 OS=Homo sapiens GN=SERPINE1 PE=1 SV=1	SERPINE1	7	8	8	4	5	3	100,0	5,83	45,060	1,29E-01
P18206	Vinculin OS=Homo sapiens GN=VCL PE=1 SV=4	VCL	8	6	6	6	6	3	100,0	5,83	123,799	4,71E-02
P68871	Hemoglobin subunit beta OS=Homo sapiens GN=HBB PE=1 SV=2	HBB	6	4	6	7	5	6	100,0	5,67	15,998	3,54E-01
P62258	14-3-3 protein epsilon OS=Homo sapiens GN=YWHAE PE=1 SV=1	YWHAE	7	7	6	5	5	4	100,0	5,67	29,174	1,94E-01
P07996	Thrombospondin-1 OS=Homo sapiens GN=THBS1 PE=1 SV=2	THBS1	2	7	3	5	7	9	100,0	5,50	129,383	4,25E-02
C9JV77	Alpha-2-HS-glycoprotein OS=Homo sapiens GN=AHSG PE=1 SV=1	AHSG	4	4	5	6	6	6	100,0	5,17	39,412	1,31E-01
P07237	Protein disulfide-isomerase OS=Homo sapiens GN=P4HB PE=1 SV=3	P4HB	4	6	7	6	4	4	100,0	5,17	57,116	9,05E-02
K7ES70	Microfibril-associated glycoprotein 4 OS=Homo sapiens GN=MFAP4 PE=1 SV=1	MFAP4	6	6	4	5	4	5	100,0	5,00	31,326	1,60E-01
P13645	Keratin, type I cytoskeletal 10 OS=Homo sapiens GN=KRT10 PE=1 SV=6	KRT10	7	8	0	0	7	7	66,7	4,83	58,827	8,22E-02
P69905	Hemoglobin subunit alpha OS=Homo sapiens GN=HBA1 PE=1 SV=2	HBA1	6	4	4	7	4	3	100,0	4,67	15,258	3,06E-01
P04179	Superoxide dismutase [Mn], mitochondrial OS=Homo sapiens GN=SOD2 PE=1 SV=2	SOD2	5	4	4	4	5	6	100,0	4,67	24,722	1,89E-01
P09486	SPARC OS=Homo sapiens GN=SPARC PE=1 SV=1	SPARC	3	3	5	5	6	6	100,0	4,67	34,632	1,35E-01
P04406	Glyceraldehyde-3-phosphate dehydrogenase OS=Homo sapiens GN=GAPDH PE=1 SV=	GAPDH	6	5	4	5	3	5	100,0	4,67	36,053	1,29E-01
P01019	Angiotensinogen OS=Homo sapiens GN=AGT PE=1 SV=1	AGT	7	5	5	3	4	4	100,0	4,67	53,154	8,78E-02
O43707	Alpha-actinin-4 OS=Homo sapiens GN=ACTN4 PE=1 SV=2	ACTN4	5	9	0	7	0	7	66,7	4,67	104,854	4,45E-02
P04004	Vitronectin OS=Homo sapiens GN=VTN PE=1 SV=1	VTN	4	5	4	5	4	4	100,0	4,33	54,306	7,98E-02
P80723	Brain acid soluble protein 1 OS=Homo sapiens GN=BASP1 PE=1 SV=2	BASP1	8	7	2	4	2	2	100,0	4,17	22,693	1,84E-01
P14618	Pyruvate kinase PKM OS=Homo sapiens GN=PKM PE=1 SV=4	PKM	5	3	3	4	6	4	100,0	4,17	57,937	7,19E-02
P02461	Collagen alpha-1(III) chain OS=Homo sapiens GN=COL3A1 PE=1 SV=4	COL3A1	6	3	5	3	3	5	100,0	4,17	138,564	3,01E-02
P10909	Clusterin OS=Homo sapiens GN=CLU PE=1 SV=1	CLU	4	3	5	3	5	4	100,0	4,00	52,495	7,62E-02
P19827	Inter-alpha-trypsin inhibitor heavy chain H1 OS=Homo sapiens GN=ITIH1 PE=1 SV=3	ITIH1	6	7	0	1	4	5	83,3	3,83	101,389	3,78E-02
P08567	Pleckstrin OS=Homo sapiens GN=PLEK PE=1 SV=3	PLEK	3	5	3	4	4	3	100,0	3,67	40,125	9,14E-02
P35527	Keratin, type I cytoskeletal 9 OS=Homo sapiens GN=KRT9 PE=1 SV=3	KRT9	6	5	1	1	5	4	100,0	3,67	62,064	5,91E-02
P19652	Alpha-1-acid glycoprotein 2 OS=Homo sapiens GN=ORM2 PE=1 SV=2	ORM2	7	5	4	0	0	5	66,7	3,50	23,603	1,48E-01
P29401	Transketolase OS=Homo sapiens GN=TKT PE=1 SV=3	TKT	5	4	3	4	2	3	100,0	3,50	67,878	5,16E-02
Q8IUX7	Adipocyte enhancer-binding protein 1 OS=Homo sapiens GN=AEBP1 PE=1 SV=1	AEBP1	4	3	4	2	4	4	100,0	3,50	130,929	2,67E-02
P00325	Alcohol dehydrogenase 1B OS=Homo sapiens GN=ADH1B PE=1 SV=2	ADH1B	3	3	4	2	3	5	100,0	3,33	39,855	8,36E-02
P12110	Collagen alpha-2(VI) chain OS=Homo sapiens GN=COL6A2 PE=1 SV=4	COL6A2	2	2	4	3	6	3	100,0	3,33	108,579	3,07E-02
P11047	Laminin subunit gamma-1 OS=Homo sapiens GN=LAMC1 PE=1 SV=3	LAMC1	5	4	3	2	3	3	100,0	3,33	177,603	1,88E-02
Q12805	EGF-containing fibulin-like extracellular matrix protein 1 OS=Homo sapiens GN=EFEMP1	EFEMP1	4	4	4	3	2	2	100,0	3,17	54,641	5,80E-02
Q15063	Periostin OS=Homo sapiens GN=POSTN PE=1 SV=2	POSTN	4	4	3	4	1	3	100,0	3,17	93,314	3,39E-02
P01619	Immunoglobulin kappa variable 3-20 OS=Homo sapiens GN=IGKV3-20 PE=1 SV=2	IGKV3-20	2	4	3	1	4	4	100,0	3,00	12,557	2,39E-01
P01011	Alpha-1-antichymotrypsin OS=Homo sapiens GN=SERPINA3 PE=1 SV=2	SERPINA3	5	1	1	2	2	7	100,0	3,00	47,651	6,30E-02
P62987	Ubiquitin-60S ribosomal protein L40 OS=Homo sapiens GN=UBA52 PE=1 SV=2	UBA52	3	2	2	3	4	3	100,0	2,83	14,728	1,92E-01
P62979	Ubiquitin-40S ribosomal protein S27a OS=Homo sapiens GN=RPS27A PE=1 SV=2	RPS27A	3	2	2	3	4	3	100,0	2,83	17,965	1,58E-01
P0CG47	Polyubiquitin-B OS=Homo sapiens GN=UBB PE=1 SV=1	UBB	3	2	2	3	4	3	100,0	2,83	25,762	1,10E-01
P0CG48	Polyubiquitin-C OS=Homo sapiens GN=UBC PE=1 SV=3	UBC	3	2	2	3	4	3	100,0	2,83	77,039	3,68E-02
A0A087WT59	Transthyretin OS=Homo sapiens GN=TTR PE=1 SV=1	TTR	4	1	2	2	3	3	100,0	2,50	20,146	1,24E-01
P68363	Tubulin alpha-1B chain OS=Homo sapiens GN=TUBA1B PE=1 SV=1	TUBA1B	2	3	3	2	3	2	100,0	2,50	50,152	4,98E-02
F5H5D3	Tubulin alpha chain OS=Homo sapiens GN=TUBA1C PE=1 SV=1	TUBA1C	2	3	3	2	3	2	100,0	2,50	57,730	4,33E-02
P04196	Histidine-rich glycoprotein OS=Homo sapiens GN=HRG PE=1 SV=1	HRG	3	2	1	4	3	2	100,0	2,50	59,578	4,20E-02
P26038	Moesin OS=Homo sapiens GN=MSN PE=1 SV=3	MSN	2	2	2	5	2	2	100,0	2,50	67,820	3,69E-02
D6RGG3	Collagen alpha-1(XII) chain OS=Homo sapiens GN=COL12A1 PE=1 SV=1	COL12A1	4	1	3	3	1	3	100,0	2,50	333,202	7,50E-03
P62805	Histone H4 OS=Homo sapiens GN=HIST1H4A PE=1 SV=2	HIST1H4A	1	3	1	2	4	3	100,0	2,33	11,367	2,05E-01

P00441	Superoxide dismutase [Cu-Zn] OS=Homo sapiens GN=SOD1 PE=1 SV=2	SOD1	0	2	3	3	3	3	83,3	2,33	15,936	1,46E-01
P01033	Metalloproteinase inhibitor 1 OS=Homo sapiens GN=TIMP1 PE=1 SV=1	TIMP1	2	3	2	3	2	2	100,0	2,33	23,171	1,01E-01
O43852	Calumenin OS=Homo sapiens GN=CALU PE=1 SV=2	CALU	1	3	0	2	3	5	83,3	2,33	37,107	6,29E-02
P27797	Calreticulin OS=Homo sapiens GN=CALR PE=1 SV=1	CALR	5	5	3	0	0	1	66,7	2,33	48,142	4,85E-02
P50395	Rab GDP dissociation inhibitor beta OS=Homo sapiens GN=GDI2 PE=1 SV=2	GDI2	1	4	1	2	4	2	100,0	2,33	50,663	4,61E-02
P06396	Gelsolin OS=Homo sapiens GN=GSN PE=1 SV=1	GSN	4	6	2	1	1	0	83,3	2,33	85,698	2,72E-02
O60814	Histone H2B type 1-K OS=Homo sapiens GN=HIST1H2BK PE=1 SV=3	HIST1H2BK	1	2	2	2	2	4	100,0	2,17	13,890	1,56E-01
Q93079	Histone H2B type 1-H OS=Homo sapiens GN=HIST1H2BH PE=1 SV=3	HIST1H2BH	1	2	2	2	2	4	100,0	2,17	13,892	1,56E-01
P06899	Histone H2B type 1-J OS=Homo sapiens GN=HIST1H2BJ PE=1 SV=3	HIST1H2BJ	1	2	2	2	2	4	100,0	2,17	13,904	1,56E-01
P62807	Histone H2B type 1-C/E/F/G/I OS=Homo sapiens GN=HIST1H2BC PE=1 SV=4	HIST1H2BC	1	2	2	2	2	4	100,0	2,17	13,906	1,56E-01
P23527	Histone H2B type 1-O OS=Homo sapiens GN=HIST1H2BO PE=1 SV=3	HIST1H2BO	1	2	2	2	2	4	100,0	2,17	13,906	1,56E-01
Q8N257	Histone H2B type 3-B OS=Homo sapiens GN=HIST3H2BB PE=1 SV=3	HIST3H2BB	1	2	2	2	2	4	100,0	2,17	13,908	1,56E-01
Q16778	Histone H2B type 2-E OS=Homo sapiens GN=HIST2H2BE PE=1 SV=3	HIST2H2BE	1	2	2	2	2	4	100,0	2,17	13,920	1,56E-01
Q5QNW6	Histone H2B type 2-F OS=Homo sapiens GN=HIST2H2BF PE=1 SV=3	HIST2H2BF	1	2	2	2	2	4	100,0	2,17	13,920	1,56E-01
P58876	Histone H2B type 1-D OS=Homo sapiens GN=HIST1H2BD PE=1 SV=2	HIST1H2BD	1	2	2	2	2	4	100,0	2,17	13,936	1,55E-01
P57053	Histone H2B type F-S OS=Homo sapiens GN=H2BFS PE=1 SV=2	H2BFS	1	2	2	2	2	4	100,0	2,17	13,944	1,55E-01
P33778	Histone H2B type 1-B OS=Homo sapiens GN=HIST1H2BB PE=1 SV=2	HIST1H2BB	1	2	2	2	2	4	100,0	2,17	13,950	1,55E-01
Q99880	Histone H2B type 1-L OS=Homo sapiens GN=HIST1H2BL PE=1 SV=3	HIST1H2BL	1	2	2	2	2	4	100,0	2,17	13,952	1,55E-01
Q99879	Histone H2B type 1-M OS=Homo sapiens GN=HIST1H2BM PE=1 SV=3	HIST1H2BM	1	2	2	2	2	4	100,0	2,17	13,989	1,55E-01
U3KQK0	Histone H2B OS=Homo sapiens GN=HIST1H2BN PE=1 SV=1	HIST1H2BN	1	2	2	2	2	4	100,0	2,17	18,804	1,15E-01
P61224	Ras-related protein Rap-1b OS=Homo sapiens GN=RAP1B PE=1 SV=1	RAP1B	0	0	3	4	3	3	66,7	2,17	20,825	1,04E-01
P62834	Ras-related protein Rap-1A OS=Homo sapiens GN=RAP1A PE=1 SV=1	RAP1A	0	0	3	4	3	3	66,7	2,17	20,987	1,03E-01
P10599	Thioredoxin OS=Homo sapiens GN=TXN PE=1 SV=3	TXN	2	2	2	2	2	2	100,0	2,00	11,737	1,70E-01
P01624	Immunoglobulin kappa variable 3-15 OS=Homo sapiens GN=IGKV3-15 PE=1 SV=2	IGKV3-15	2	2	2	2	2	2	100,0	2,00	12,496	1,60E-01
A0A0C4DH90	Protein IGKV3OR2-268 (Fragment) OS=Homo sapiens GN=IGKV3OR2-268 PE=4 SV=5	IGKV3OR2-268	2	2	2	2	2	2	100,0	2,00	12,586	1,59E-01
A0A075B6H7	Protein IGKV3-7 (Fragment) OS=Homo sapiens GN=IGKV3-7 PE=1 SV=1	IGKV3-7	2	2	2	2	2	2	100,0	2,00	12,783	1,56E-01
A0A0C4DH55	Protein IGKV3D-7 (Fragment) OS=Homo sapiens GN=IGKV3D-7 PE=4 SV=5	IGKV3D-7	2	2	2	2	2	2	100,0	2,00	13,148	1,52E-01
P37802	Transgelin-2 OS=Homo sapiens GN=TAGLN2 PE=1 SV=3	TAGLN2	2	2	2	2	2	2	100,0	2,00	22,391	8,93E-02
B9A064	Immunoglobulin lambda-like polypeptide 5 OS=Homo sapiens GN=IGLL5 PE=2 SV=2	IGLL5	0	0	0	0	7	5	33,3	2,00	23,063	8,67E-02
Q92520	Protein FAM3C OS=Homo sapiens GN=FAM3C PE=1 SV=1	FAM3C	2	2	2	2	2	2	100,0	2,00	24,680	8,10E-02
P31946	14-3-3 protein beta/alpha OS=Homo sapiens GN=YWHAB PE=1 SV=3	YWHAB	5	4	2	1	0	0	66,7	2,00	28,082	7,12E-02
P07195	L-lactate dehydrogenase B chain OS=Homo sapiens GN=LDHB PE=1 SV=2	LDHB	0	3	2	3	2	2	83,3	2,00	36,638	5,46E-02
P21810	Biglycan OS=Homo sapiens GN=BGN PE=1 SV=2	BGN	2	2	2	2	2	2	100,0	2,00	41,654	4,80E-02
A0A0A0MTS2	Glucose-6-phosphate isomerase (Fragment) OS=Homo sapiens GN=GPI PE=1 SV=1	GPI	2	2	1	2	3	2	100,0	2,00	64,825	3,09E-02
P15144	Aminopeptidase N OS=Homo sapiens GN=ANPEP PE=1 SV=4	ANPEP	0	1	2	3	3	3	83,3	2,00	109,540	1,83E-02
P06703	Protein S100-A6 OS=Homo sapiens GN=S100A6 PE=1 SV=1	S100A6	1	2	2	2	2	2	100,0	1,83	10,180	1,80E-01
S4R460	Protein IGHV3OR16-9 OS=Homo sapiens GN=IGHV3OR16-9 PE=1 SV=2	IGHV3OR16-9	1	2	2	2	2	2	100,0	1,83	10,429	1,76E-01
A0A087WW89	Protein IGHV3-72 OS=Homo sapiens GN=IGHV3-72 PE=1 SV=1	IGHV3-72	1	2	2	2	2	2	100,0	1,83	13,203	1,39E-01
Q96KK5	Histone H2A type 1-H OS=Homo sapiens GN=HIST1H2AH PE=1 SV=3	HIST1H2AH	2	2	2	1	2	2	100,0	1,83	13,906	1,32E-01
Q99878	Histone H2A type 1-J OS=Homo sapiens GN=HIST1H2AJ PE=1 SV=3	HIST1H2AJ	2	2	2	1	2	2	100,0	1,83	13,936	1,32E-01
Q16777	Histone H2A type 2-C OS=Homo sapiens GN=HIST2H2AC PE=1 SV=4	HIST2H2AC	2	2	2	1	2	2	100,0	1,83	13,988	1,31E-01
Q9BTM1	Histone H2A.J OS=Homo sapiens GN=H2AFJ PE=1 SV=1	H2AFJ	2	2	2	1	2	2	100,0	1,83	14,019	1,31E-01
P0C0S8	Histone H2A type 1 OS=Homo sapiens GN=HIST1H2AG PE=1 SV=2	HIST1H2AG	2	2	2	1	2	2	100,0	1,83	14,091	1,30E-01
Q6FI13	Histone H2A type 2-A OS=Homo sapiens GN=HIST2H2AA3 PE=1 SV=3	HIST2H2AA3	2	2	2	1	2	2	100,0	1,83	14,095	1,30E-01
Q93077	Histone H2A type 1-C OS=Homo sapiens GN=HIST1H2AC PE=1 SV=3	HIST1H2AC	2	2	2	1	2	2	100,0	1,83	14,105	1,30E-01
P20671	Histone H2A type 1-D OS=Homo sapiens GN=HIST1H2AD PE=1 SV=2	HIST1H2AD	2	2	2	1	2	2	100,0	1,83	14,107	1,30E-01
Q7L7L0	Histone H2A type 3 OS=Homo sapiens GN=HIST3H2A PE=1 SV=3	HIST3H2A	2	2	2	1	2	2	100,0	1,83	14,121	1,30E-01
P04908	Histone H2A type 1-B/E OS=Homo sapiens GN=HIST1H2AB PE=1 SV=2	HIST1H2AB	2	2	2	1	2	2	100,0	1,83	14,135	1,30E-01
A0A0U1RRH7	Histone H2A OS=Homo sapiens PE=3 SV=1	HIST1H3D	2	2	2	1	2	2	100,0	1,83	18,553	9,88E-02
P07093	Glia-derived nexin OS=Homo sapiens GN=SERPINE2 PE=1 SV=1	SERPINE2	2	1	2	2	2	2	100,0	1,83	44,002	4,17E-02
P50454	Serpin H1 OS=Homo sapiens GN=SERPINH1 PE=1 SV=2	SERPINH1	2	3	2	1	2	1	100,0	1,83	46,441	3,95E-02
P00734	Prothrombin OS=Homo sapiens GN=F2 PE=1 SV=2	F2	0	1	3	3	2	2	83,3	1,83	70,037	2,62E-02
Q13201	Multimerin-1 OS=Homo sapiens GN=MMRN1 PE=1 SV=3	MMRN1	0	2	2	1	2	4	83,3	1,83	138,110	1,33E-02
P04433	Immunoglobulin kappa variable 3-11 OS=Homo sapiens GN=IGKV3-11 PE=1 SV=1	IGKV3-11	1	2	1	2	2	2	100,0	1,67	12,575	1,33E-01
A0A0A0MRZ8	Protein IGKV3D-11 (Fragment) OS=Homo sapiens GN=IGKV3D-11 PE=4 SV=6	IGKV3D-11	1	2	1	2	2	2	100,0	1,67	12,625	1,32E-01
Q9BRK5	45 kDa calcium-binding protein OS=Homo sapiens GN=SDF4 PE=1 SV=1	SDF4	3	1	0	2	2	2	83,3	1,67	41,807	3,99E-02
Q562R1	Beta-actin-like protein 2 OS=Homo sapiens GN=ACTBL2 PE=1 SV=2	ACTBL2	5	0	0	0	5	0	33,3	1,67	42,003	3,97E-02

Q71U36	Tubulin alpha-1A chain OS=Homo sapiens GN=TUBA1A PE=1 SV=1	TUBA1A	0	0	3	2	3	2	66,7	1,67	50,136	3,32E-02
P35241	Radixin OS=Homo sapiens GN=RDX PE=1 SV=1	RDX	2	2	2	0	2	2	83,3	1,67	68,564	2,43E-02
E7EQR4	Ezrin OS=Homo sapiens GN=EZR PE=1 SV=3	EZR	2	2	2	0	2	2	83,3	1,67	69,372	2,40E-02
P05386	60S acidic ribosomal protein P1 OS=Homo sapiens GN=RPLP1 PE=1 SV=1	RPLP1	2	2	2	0	1	2	83,3	1,50	11,514	1,30E-01
A0A075B6P5	Immunoglobulin kappa variable 2-28 OS=Homo sapiens GN=IGKV2-28 PE=3 SV=1	IGKV2-28	0	2	2	2	1	2	83,3	1,50	12,957	1,16E-01
A0A075B6S2	Protein IGKV2D-29 (Fragment) OS=Homo sapiens GN=IGKV2D-29 PE=1 SV=1	IGKV2D-29	0	2	2	2	1	2	83,3	1,50	13,143	1,14E-01
P06310	Immunoglobulin kappa variable 2-30 OS=Homo sapiens GN=IGKV2-30 PE=3 SV=2	IGKV2-30	0	2	2	2	1	2	83,3	1,50	13,185	1,14E-01
A0A075B6S6	Immunoglobulin kappa variable 2D-30 OS=Homo sapiens GN=IGKV2D-30 PE=3 SV=1	IGKV2D-30	0	2	2	2	1	2	83,3	1,50	13,215	1,14E-01
A0A0A0MRZ7	Protein IGKV2D-26 (Fragment) OS=Homo sapiens GN=IGKV2D-26 PE=4 SV=1	IGKV2D-26	0	2	2	2	1	2	83,3	1,50	13,297	1,13E-01
A0A087WW87	Protein IGKV2-40 (Fragment) OS=Homo sapiens GN=IGKV2-40 PE=1 SV=1	IGKV2-40	0	2	2	2	1	2	83,3	1,50	13,310	1,13E-01
P01614	Immunoglobulin kappa variable 2D-40 OS=Homo sapiens GN=IGKV2D-40 PE=1 SV=2	IGKV2D-40	0	2	2	2	1	2	83,3	1,50	13,310	1,13E-01
P01860	Ig gamma-3 chain C region OS=Homo sapiens GN=IGHG3 PE=1 SV=2	IGHG3	9	0	0	0	0	0	16,7	1,50	41,287	3,63E-02
P78539	Sushi repeat-containing protein SRPX OS=Homo sapiens GN=SRPX PE=1 SV=1	SRPX	0	2	2	1	2	2	83,3	1,50	51,572	2,91E-02
P55072	Transitional endoplasmic reticulum ATPase OS=Homo sapiens GN=VCP PE=1 SV=4	VCP	2	1	2	2	1	1	100,0	1,50	89,322	1,68E-02
Q14624	Inter-alpha-trypsin inhibitor heavy chain H4 OS=Homo sapiens GN=ITIH4 PE=1 SV=4	ITIH4	3	1	1	2	1	1	100,0	1,50	103,357	1,45E-02
P35442	Thrombospondin-2 OS=Homo sapiens GN=THBS2 PE=1 SV=2	THBS2	3	3	1	0	1	1	83,3	1,50	129,991	1,15E-02
P02776	Platelet factor 4 OS=Homo sapiens GN=PF4 PE=1 SV=2	PF4	0	1	2	1	2	2	83,3	1,33	10,845	1,23E-01
P10720	Platelet factor 4 variant OS=Homo sapiens GN=PF4V1 PE=1 SV=1	PF4V1	0	1	2	1	2	2	83,3	1,33	11,553	1,15E-01
P61769	Beta-2-microglobulin OS=Homo sapiens GN=B2M PE=1 SV=1	B2M	1	2	1	1	2	1	100,0	1,33	13,715	9,72E-02
P16035	Metalloproteinase inhibitor 2 OS=Homo sapiens GN=TIMP2 PE=1 SV=2	TIMP2	0	0	2	2	2	2	66,7	1,33	24,399	5,46E-02
P02649	Apolipoprotein E OS=Homo sapiens GN=APOE PE=1 SV=1	APOE	1	1	0	2	2	2	83,3	1,33	36,154	3,69E-02
Q14CN4	Keratin, type II cytoskeletal 72 OS=Homo sapiens GN=KRT72 PE=1 SV=2	KRT72	2	2	2	1	1	0	83,3	1,33	55,877	2,39E-02
Q3SY84	Keratin, type II cytoskeletal 71 OS=Homo sapiens GN=KRT71 PE=1 SV=3	KRT71	2	2	2	1	1	0	83,3	1,33	57,292	2,33E-02
Q86Y46	Keratin, type II cytoskeletal 73 OS=Homo sapiens GN=KRT73 PE=1 SV=1	KRT73	2	2	2	1	1	0	83,3	1,33	58,923	2,26E-02
F8W1S1	Keratin, type II cytoskeletal 74 OS=Homo sapiens GN=KRT74 PE=1 SV=1	KRT74	2	2	2	1	1	0	83,3	1,33	59,407	2,24E-02
P00751	Complement factor B OS=Homo sapiens GN=CFB PE=1 SV=2	CFB	4	3	1	0	0	0	50,0	1,33	85,533	1,56E-02
P00747	Plasminogen OS=Homo sapiens GN=PLG PE=1 SV=2	PLG	1	3	2	2	0	0	66,7	1,33	90,569	1,47E-02
B4E1Z4	Uncharacterized protein OS=Homo sapiens PE=1 SV=1	C2	4	3	1	0	0	0	50,0	1,33	140,943	9,46E-03
Q14767	Latent-transforming growth factor beta-binding protein 2 OS=Homo sapiens GN=LTBP2 F	LTBP2	2	1	1	2	1	1	100,0	1,33	195,052	6,84E-03
P09382	Galectin-1 OS=Homo sapiens GN=LGALS1 PE=1 SV=2	LGALS1	0	1	2	2	1	1	83,3	1,17	14,716	7,93E-02
P07737	Profilin-1 OS=Homo sapiens GN=PFN1 PE=1 SV=2	PFN1	2	0	2	2	1	0	66,7	1,17	15,054	7,75E-02
A0A087WWU8	Tropomyosin alpha-3 chain OS=Homo sapiens GN=TPM3 PE=1 SV=1	TPM3	3	0	0	4	0	0	33,3	1,17	26,421	4,42E-02
Q5HYB6	Epididymis luminal protein 189 OS=Homo sapiens GN=DKFZp686J1372 PE=1 SV=1	DKFZp686J1372	3	0	0	4	0	0	33,3	1,17	27,176	4,29E-02
Q08380	Galectin-3-binding protein OS=Homo sapiens GN=LGALS3BP PE=1 SV=1	LGALS3BP	1	0	1	2	2	1	83,3	1,17	65,331	1,79E-02
P55268	Laminin subunit beta-2 OS=Homo sapiens GN=LAMB2 PE=1 SV=2	LAMB2	3	1	1	1	1	0	83,3	1,17	195,981	5,95E-03
P31949	Protein S100-A11 OS=Homo sapiens GN=S100A11 PE=1 SV=2	S100A11	0	1	0	2	2	1	66,7	1,00	11,740	8,52E-02
P20618	Proteasome subunit beta type-1 OS=Homo sapiens GN=PSMB1 PE=1 SV=2	PSMB1	1	0	1	1	1	2	83,3	1,00	26,489	3,78E-02
P29966	Myristoylated alanine-rich C-kinase substrate OS=Homo sapiens GN=MARCKS PE=1 SV	MARCKS	2	0	2	2	0	0	50,0	1,00	31,555	3,17E-02
Q969T7	7-methylguanosine phosphate-specific 5'-nucleotidase OS=Homo sapiens GN=NT5C3B I	NT5C3B	0	3	0	0	1	2	50,0	1,00	34,389	2,91E-02
P02760	Protein AMBP OS=Homo sapiens GN=AMBP PE=1 SV=1	AMBP	1	2	0	0	2	1	66,7	1,00	38,999	2,56E-02
J3KPS3	Fructose-bisphosphate aldolase OS=Homo sapiens GN=ALDOA PE=1 SV=1	ALDOA	0	0	1	2	1	2	66,7	1,00	39,817	2,51E-02
P00326	Alcohol dehydrogenase 1C OS=Homo sapiens GN=ADH1C PE=1 SV=2	ADH1C	2	1	1	2	0	0	66,7	1,00	39,868	2,51E-02
P11142	Heat shock cognate 71 kDa protein OS=Homo sapiens GN=HSPA8 PE=1 SV=1	HSPA8	0	0	0	3	2	1	50,0	1,00	70,898	1,41E-02
P11021	78 kDa glucose-regulated protein OS=Homo sapiens GN=HSPA5 PE=1 SV=2	HSPA5	0	0	3	0	2	1	50,0	1,00	72,333	1,38E-02
P53675	Clathrin heavy chain 2 OS=Homo sapiens GN=CLTCL1 PE=1 SV=2	CLTCL1	0	1	2	2	1	0	66,7	1,00	187,030	5,35E-03
A0A087WVQ6	Clathrin heavy chain OS=Homo sapiens GN=CLTC PE=1 SV=1	CLTC	0	1	2	2	1	0	66,7	1,00	192,058	5,21E-03
P27348	14-3-3 protein theta OS=Homo sapiens GN=YWHAQ PE=1 SV=1	YWHAQ	2	3	0	0	0	0	33,3	0,83	27,764	3,00E-02
P61981	14-3-3 protein gamma OS=Homo sapiens GN=YWHAG PE=1 SV=2	YWHAG	3	0	1	1	0	0	50,0	0,83	28,303	2,94E-02
P27105	Erythrocyte band 7 integral membrane protein OS=Homo sapiens GN=STOM PE=1 SV=3	STOM	1	0	1	2	1	0	66,7	0,83	31,731	2,63E-02
Q15113	Procollagen C-endopeptidase enhancer 1 OS=Homo sapiens GN=PCOLCE PE=1 SV=2	PCOLCE	2	1	2	0	0	0	50,0	0,83	47,972	1,74E-02
Q16881	Thioredoxin reductase 1, cytoplasmic OS=Homo sapiens GN=TXNRD1 PE=1 SV=3	TXNRD1	0	1	1	1	1	1	83,3	0,83	70,906	1,18E-02
P24821	Tenascin OS=Homo sapiens GN=TNC PE=1 SV=3	TNC	0	0	1	2	0	2	50,0	0,83	240,853	3,46E-03
H7BZJ8	Zinc-alpha-2-glycoprotein (Fragment) OS=Homo sapiens GN=AZGP1 PE=1 SV=1	AZGP1	0	0	0	2	1	1	50,0	0,67	9,709	6,87E-02
P62937	Peptidyl-prolyl cis-trans isomerase A OS=Homo sapiens GN=PPIA PE=1 SV=2	PPIA	1	0	2	1	0	0	50,0	0,67	18,012	3,70E-02
B8ZZZ5	PH and SEC7 domain-containing protein 4 (Fragment) OS=Homo sapiens GN=PSD4 PE	PSD4	2	0	0	0	0	2	33,3	0,67	19,927	3,35E-02
C9IZN3	Actin-related protein 3C (Fragment) OS=Homo sapiens GN=ACTR3C PE=3 SV=1	ACTR3C	1	1	1	0	0	1	66,7	0,67	24,320	2,74E-02
P40926	Malate dehydrogenase, mitochondrial OS=Homo sapiens GN=MDH2 PE=1 SV=3	MDH2	0	0	1	0	2	1	50,0	0,67	35,503	1,88E-02

E9PF18	Hydroxyacyl-coenzyme A dehydrogenase, mitochondrial OS=Homo sapiens GN=HADH F	HADH	0	1	1	1	1	0	66,7	0,67	42,123	1,58E-02
P61158	Actin-related protein 3 OS=Homo sapiens GN=ACTR3 PE=1 SV=3	ACTR3	1	1	1	0	0	1	66,7	0,67	47,371	1,41E-02
Q9P1U1	Actin-related protein 3B OS=Homo sapiens GN=ACTR3B PE=2 SV=1	ACTR3B	1	1	1	0	0	1	66,7	0,67	47,608	1,40E-02
P07437	Tubulin beta chain OS=Homo sapiens GN=TUBB PE=1 SV=2	TUBB	1	0	2	1	0	0	50,0	0,67	49,671	1,34E-02
P68371	Tubulin beta-4B chain OS=Homo sapiens GN=TUBB4B PE=1 SV=1	TUBB4B	1	0	2	1	0	0	50,0	0,67	49,831	1,34E-02
Q13885	Tubulin beta-2A chain OS=Homo sapiens GN=TUBB2A PE=1 SV=1	TUBB2A	1	0	2	1	0	0	50,0	0,67	49,907	1,34E-02
Q9BVA1	Tubulin beta-2B chain OS=Homo sapiens GN=TUBB2B PE=1 SV=1	TUBB2B	1	0	2	1	0	0	50,0	0,67	49,953	1,33E-02
P02545	Prelamin-A/C OS=Homo sapiens GN=LMNA PE=1 SV=1	LMNA	1	1	1	1	0	0	66,7	0,67	74,139	8,99E-03
P98095	Fibulin-2 OS=Homo sapiens GN=FBLN2 PE=1 SV=2	FBLN2	0	2	1	1	0	0	50,0	0,67	126,573	5,27E-03
P01031	Complement C5 OS=Homo sapiens GN=C5 PE=1 SV=4	C5	2	2	0	0	0	0	33,3	0,67	188,305	3,54E-03
Q4LDE5	Sushi, von Willebrand factor type A, EGF and pentraxin domain-containing protein 1 OS=	SVEP1	1	2	0	0	1	0	50,0	0,67	390,170	1,71E-03
Q9H299	SH3 domain-binding glutamic acid-rich-like protein 3 OS=Homo sapiens GN=SH3BGRL3	SH3BGRL3	0	0	0	0	1	2	33,3	0,50	10,438	4,79E-02
A0A0C4DH25	Immunoglobulin kappa variable 3-20 OS=Homo sapiens GN=IGKV3D-20 PE=3 SV=1	IGKV3D-20	2	0	0	1	0	0	33,3	0,50	12,515	4,00E-02
P01602	Immunoglobulin kappa variable 1-5 OS=Homo sapiens GN=IGKV1-5 PE=1 SV=2	IGKV1-5	0	2	1	0	0	0	33,3	0,50	12,782	3,91E-02
P06312	Immunoglobulin kappa variable 4-1 OS=Homo sapiens GN=IGKV4-1 PE=1 SV=1	IGKV4-1	0	0	0	0	1	2	33,3	0,50	13,380	3,74E-02
D6RHJ6	Immunoglobulin J chain (Fragment) OS=Homo sapiens GN=JCHAIN PE=1 SV=1	JCHAIN	2	0	1	0	0	0	33,3	0,50	17,679	2,83E-02
P43235	Cathepsin K OS=Homo sapiens GN=CTSK PE=1 SV=1	CTSK	1	1	1	0	0	0	50,0	0,50	36,966	1,35E-02
P07711	Cathepsin L1 OS=Homo sapiens GN=CTSL PE=1 SV=2	CTSL	2	0	1	0	0	0	33,3	0,50	37,564	1,33E-02
P07355	Annexin A2 OS=Homo sapiens GN=ANXA2 PE=1 SV=2	ANXA2	0	0	1	0	0	2	33,3	0,50	38,604	1,30E-02
A6NMY6	Putative annexin A2-like protein OS=Homo sapiens GN=ANXA2P2 PE=5 SV=2	ANXA2P2	0	0	1	0	0	2	33,3	0,50	38,659	1,29E-02
P48741	Putative heat shock 70 kDa protein 7 OS=Homo sapiens GN=HSPA7 PE=5 SV=2	HSPA7	0	1	0	0	0	2	33,3	0,50	40,244	1,24E-02
P06727	Apolipoprotein A-IV OS=Homo sapiens GN=APOA4 PE=1 SV=3	APOA4	1	1	0	0	0	1	50,0	0,50	45,399	1,10E-02
P68104	Elongation factor 1-alpha 1 OS=Homo sapiens GN=EEF1A1 PE=1 SV=1	EEF1A1	2	0	0	0	1	0	33,3	0,50	50,141	9,97E-03
Q5VTE0	Putative elongation factor 1-alpha-like 3 OS=Homo sapiens GN=EEF1A1P5 PE=5 SV=1	EEF1A1P5	2	0	0	0	1	0	33,3	0,50	50,185	9,96E-03
Q01518	Adenylyl cyclase-associated protein 1 OS=Homo sapiens GN=CAP1 PE=1 SV=5	CAP1	1	1	0	0	1	0	50,0	0,50	51,901	9,63E-03
P08697	Alpha-2-antiplasmin OS=Homo sapiens GN=SERPINF2 PE=1 SV=3	SERPINF2	1	0	1	1	0	0	50,0	0,50	54,566	9,16E-03
P40227	T-complex protein 1 subunit zeta OS=Homo sapiens GN=CCT6A PE=1 SV=3	CCT6A	0	1	0	1	1	0	50,0	0,50	58,024	8,62E-03
P36871	Phosphoglucomutase-1 OS=Homo sapiens GN=PGM1 PE=1 SV=3	PGM1	0	0	1	1	1	0	50,0	0,50	61,449	8,14E-03
P54652	Heat shock-related 70 kDa protein 2 OS=Homo sapiens GN=HSPA2 PE=1 SV=1	HSPA2	0	0	0	0	2	1	33,3	0,50	70,021	7,14E-03
P34931	Heat shock 70 kDa protein 1-like OS=Homo sapiens GN=HSPA1L PE=1 SV=2	HSPA1L	0	0	0	0	2	1	33,3	0,50	70,375	7,10E-03
P17066	Heat shock 70 kDa protein 6 OS=Homo sapiens GN=HSPA6 PE=1 SV=2	HSPA6	0	1	0	0	0	2	33,3	0,50	71,028	7,04E-03
O00391	Sulfhydryl oxidase 1 OS=Homo sapiens GN=QSOX1 PE=1 SV=3	QSOX1	0	0	1	1	0	1	50,0	0,50	82,578	6,05E-03
Q9Y6C2	EMILIN-1 OS=Homo sapiens GN=EMILIN1 PE=1 SV=2	EMILIN1	1	1	0	0	1	0	50,0	0,50	106,667	4,69E-03
P35579	Myosin-9 OS=Homo sapiens GN=MYH9 PE=1 SV=4	MYH9	1	0	0	0	0	2	33,3	0,50	226,532	2,21E-03
P62314	Small nuclear ribonucleoprotein Sm D1 OS=Homo sapiens GN=SNRPD1 PE=1 SV=1	SNRPD1	0	0	2	0	0	0	16,7	0,33	13,282	2,51E-02
P41222	Prostaglandin-H2 D-isomerase OS=Homo sapiens GN=PTGDS PE=1 SV=1	PTGDS	2	0	0	0	0	0	16,7	0,33	21,029	1,59E-02
E9PK25	Cofilin-1 OS=Homo sapiens GN=CFL1 PE=1 SV=1	CFL1	1	1	0	0	0	0	33,3	0,33	22,728	1,47E-02
P02743	Serum amyloid P-component OS=Homo sapiens GN=APCS PE=1 SV=2	APCS	2	0	0	0	0	0	16,7	0,33	25,387	1,31E-02
B7Z6Z4	Myosin light polypeptide 6 OS=Homo sapiens GN=MYL6 PE=1 SV=1	MYL6	0	1	0	0	0	1	33,3	0,33	26,707	1,25E-02
Q15404	Ras suppressor protein 1 OS=Homo sapiens GN=RSU1 PE=1 SV=3	RSU1	0	2	0	0	0	0	16,7	0,33	31,540	1,06E-02
P07327	Alcohol dehydrogenase 1A OS=Homo sapiens GN=ADH1A PE=1 SV=2	ADH1A	0	0	0	2	0	0	16,7	0,33	39,859	8,36E-03
P18464	HLA class I histocompatibility antigen, B-51 alpha chain OS=Homo sapiens GN=HLA-B F	HLA-B	0	0	0	1	1	0	33,3	0,33	40,566	8,22E-03
Q9BYZ2	L-lactate dehydrogenase A-like 6B OS=Homo sapiens GN=LDHAL6B PE=1 SV=3	LDHAL6B	0	0	0	2	0	0	16,7	0,33	41,943	7,95E-03
P36222	Chitinase-3-like protein 1 OS=Homo sapiens GN=CHI3L1 PE=1 SV=2	CHI3L1	0	2	0	0	0	0	16,7	0,33	42,625	7,82E-03
P54727	UV excision repair protein RAD23 homolog B OS=Homo sapiens GN=RAD23B PE=1 SV	RAD23B	0	1	1	0	0	0	33,3	0,33	43,171	7,72E-03
A2BF26	HLA class I histocompatibility antigen, Cw-6 alpha chain OS=Homo sapiens GN=HLA-C F	HLA-C	0	0	0	1	1	0	33,3	0,33	44,532	7,49E-03
Q9H4B7	Tubulin beta-1 chain OS=Homo sapiens GN=TUBB1 PE=1 SV=1	TUBB1	0	0	1	1	0	0	33,3	0,33	50,327	6,62E-03
Q13509	Tubulin beta-3 chain OS=Homo sapiens GN=TUBB3 PE=1 SV=2	TUBB3	1	0	0	1	0	0	33,3	0,33	50,433	6,61E-03
P24347	Stromelysin-3 OS=Homo sapiens GN=MMP11 PE=1 SV=3	MMP11	2	0	0	0	0	0	16,7	0,33	54,590	6,11E-03
P05546	Heparin cofactor 2 OS=Homo sapiens GN=SERPIND1 PE=1 SV=3	SERPIND1	0	0	1	0	0	1	33,3	0,33	57,071	5,84E-03
O95800	Probable G-protein coupled receptor 75 OS=Homo sapiens GN=GPR75 PE=1 SV=1	GPR75	0	1	1	0	0	0	33,3	0,33	59,359	5,62E-03
P22792	Carboxypeptidase N subunit 2 OS=Homo sapiens GN=CPN2 PE=1 SV=3	CPN2	0	1	0	0	1	0	33,3	0,33	60,557	5,50E-03
Q15942	Zyxin OS=Homo sapiens GN=ZYX PE=1 SV=1	ZYX	1	0	1	0	0	0	33,3	0,33	61,277	5,44E-03
Q14956	Transmembrane glycoprotein NMB OS=Homo sapiens GN=GPNMB PE=1 SV=2	GPNMB	1	1	0	0	0	0	33,3	0,33	63,923	5,21E-03
Q9C0D0	Phosphatase and actin regulator 1 OS=Homo sapiens GN=PHACTR1 PE=1 SV=3	PHACTR1	0	0	0	1	0	1	33,3	0,33	66,308	5,03E-03
P0DMV9	Heat shock 70 kDa protein 1B OS=Homo sapiens GN=HSPA1B PE=1 SV=1	HSPA1B	0	0	0	0	2	0	16,7	0,33	70,052	4,76E-03
P13639	Elongation factor 2 OS=Homo sapiens GN=EEF2 PE=1 SV=4	EEF2	1	1	0	0	0	0	33,3	0,33	95,338	3,50E-03

E7EUF1	Ectonucleotide pyrophosphatase/phosphodiesterase family member 2 OS=Homo sapiens	ENPP2	2	0	0	0	0	0	16,7	0,33	101,553	3,28E-03
Q9P0K7	Ankycorbin OS=Homo sapiens GN=RAI14 PE=1 SV=2	RAI14	0	0	0	1	0	1	33,3	0,33	110,041	3,03E-03
A0A087WXW9	Collagen alpha-1(V) chain OS=Homo sapiens GN=COL5A1 PE=1 SV=1	COL5A1	0	0	1	0	0	1	33,3	0,33	183,653	1,82E-03
P82921	28S ribosomal protein S21, mitochondrial OS=Homo sapiens GN=MRPS21 PE=1 SV=2	MRPS21	0	0	1	0	0	0	16,7	0,17	10,742	1,55E-02
P01717	Immunoglobulin lambda variable 3-25 OS=Homo sapiens GN=IGLV3-25 PE=1 SV=2	IGLV3-25	0	0	1	0	0	0	16,7	0,17	12,011	1,39E-02
Q8WWY7	WAP four-disulfide core domain protein 12 OS=Homo sapiens GN=WFDC12 PE=2 SV=1	WFDC12	0	1	0	0	0	0	16,7	0,17	12,050	1,38E-02
P01718	Immunoglobulin lambda variable 3-27 OS=Homo sapiens GN=IGLV3-27 PE=1 SV=2	IGLV3-27	0	0	1	0	0	0	16,7	0,17	12,165	1,37E-02
A0A075B6K0	HCG2041221 (Fragment) OS=Homo sapiens GN=IGLV3-16 PE=1 SV=1	IGLV3-16	0	0	1	0	0	0	16,7	0,17	12,466	1,34E-02
P01764	Immunoglobulin heavy variable 3-23 OS=Homo sapiens GN=IGHV3-23 PE=1 SV=2	IGHV3-23	1	0	0	0	0	0	16,7	0,17	12,582	1,32E-02
A0A0C4DH42	Protein IGHV3-66 (Fragment) OS=Homo sapiens GN=IGHV3-66 PE=4 SV=1	IGHV3-66	1	0	0	0	0	0	16,7	0,17	12,698	1,31E-02
P01767	Immunoglobulin heavy variable 3-53 OS=Homo sapiens GN=IGHV3-53 PE=1 SV=2	IGHV3-53	1	0	0	0	0	0	16,7	0,17	12,770	1,31E-02
A0A0B4J1X5	Protein IGHV3-74 (Fragment) OS=Homo sapiens GN=IGHV3-74 PE=1 SV=1	IGHV3-74	1	0	0	0	0	0	16,7	0,17	12,840	1,30E-02
A0A075B7B8	Protein IGHV3OR16-12 (Fragment) OS=Homo sapiens GN=IGHV3OR16-12 PE=1 SV=1	IGHV3OR16-12	1	0	0	0	0	0	16,7	0,17	12,875	1,29E-02
A0A075B7E8	Protein IGHV3OR16-13 (Fragment) OS=Homo sapiens GN=IGHV3OR16-13 PE=4 SV=1	IGHV3OR16-13	1	0	0	0	0	0	16,7	0,17	12,904	1,29E-02
P01825	Immunoglobulin heavy variable 4-59 OS=Homo sapiens GN=IGHV4-59 PE=1 SV=2	IGHV4-59	0	0	0	0	0	1	16,7	0,17	12,936	1,29E-02
P01768	Immunoglobulin heavy variable 3-30 OS=Homo sapiens GN=IGHV3-30 PE=1 SV=2	IGHV3-30	1	0	0	0	0	0	16,7	0,17	12,947	1,29E-02
A0A0C4DH41	Protein IGHV4-61 (Fragment) OS=Homo sapiens GN=IGHV4-61 PE=4 SV=1	IGHV4-61	0	0	0	0	0	1	16,7	0,17	13,066	1,28E-02
P01772	Immunoglobulin heavy variable 3-33 OS=Homo sapiens GN=IGHV3-33 PE=1 SV=2	IGHV3-33	1	0	0	0	0	0	16,7	0,17	13,074	1,27E-02
P06331	Immunoglobulin heavy variable 4-34 OS=Homo sapiens GN=IGHV4-34 PE=1 SV=2	IGHV4-34	0	0	0	0	0	1	16,7	0,17	13,815	1,21E-02
P01824	Immunoglobulin heavy variable 4-39 OS=Homo sapiens GN=IGHV4-39 PE=1 SV=2	IGHV4-39	0	0	0	0	0	1	16,7	0,17	13,917	1,20E-02
Q96A08	Histone H2B type 1-A OS=Homo sapiens GN=HIST1H2BA PE=1 SV=3	HIST1H2BA	1	0	0	0	0	0	16,7	0,17	14,167	1,18E-02
O60361	Putative nucleoside diphosphate kinase OS=Homo sapiens GN=NME2P1 PE=5 SV=1	NME2P1	0	1	0	0	0	0	16,7	0,17	15,529	1,07E-02
P22392	Nucleoside diphosphate kinase B OS=Homo sapiens GN=NME2 PE=1 SV=1	NME2	0	1	0	0	0	0	16,7	0,17	17,298	9,64E-03
Q969E4	Transcription elongation factor A protein-like 3 OS=Homo sapiens GN=TCEAL3 PE=1 SV	TCEAL3	1	0	0	0	0	0	16,7	0,17	22,502	7,41E-03
P09211	Glutathione S-transferase P OS=Homo sapiens GN=GSTP1 PE=1 SV=2	GSTP1	0	0	1	0	0	0	16,7	0,17	23,356	7,14E-03
P23284	Peptidyl-prolyl cis-trans isomerase B OS=Homo sapiens GN=PPIB PE=1 SV=2	PPIB	0	1	0	0	0	0	16,7	0,17	23,743	7,02E-03
P08294	Extracellular superoxide dismutase [Cu-Zn] OS=Homo sapiens GN=SOD3 PE=1 SV=2	SOD3	1	0	0	0	0	0	16,7	0,17	25,851	6,45E-03
P22692	Insulin-like growth factor-binding protein 4 OS=Homo sapiens GN=IGFBP4 PE=1 SV=2	IGFBP4	0	0	1	0	0	0	16,7	0,17	27,934	5,97E-03
G3V5Z7	Proteasome subunit alpha type OS=Homo sapiens GN=PSMA6 PE=1 SV=1	PSMA6	1	0	0	0	0	0	16,7	0,17	28,147	5,92E-03
Q16270	Insulin-like growth factor-binding protein 7 OS=Homo sapiens GN=IGFBP7 PE=1 SV=1	IGFBP7	0	0	0	0	0	1	16,7	0,17	29,130	5,72E-03
Q32Q12	Nucleoside diphosphate kinase OS=Homo sapiens GN=NME1-NME2 PE=1 SV=1	NME1-NME2	0	1	0	0	0	0	16,7	0,17	32,642	5,11E-03
Q99598	Translin-associated protein X OS=Homo sapiens GN=TSNAX PE=1 SV=1	TSNAX	0	1	0	0	0	0	16,7	0,17	33,112	5,03E-03
P08758	Annexin A5 OS=Homo sapiens GN=ANXA5 PE=1 SV=2	ANXA5	0	0	1	0	0	0	16,7	0,17	35,937	4,64E-03
P40925	Malate dehydrogenase, cytoplasmic OS=Homo sapiens GN=MDH1 PE=1 SV=4	MDH1	0	0	0	1	0	0	16,7	0,17	36,426	4,58E-03
M0QXB4	Coatomer protein complex, subunit epsilon, isoform CRA_g OS=Homo sapiens GN=COF	COPE	0	0	1	0	0	0	16,7	0,17	36,924	4,51E-03
Q02985	Complement factor H-related protein 3 OS=Homo sapiens GN=CFHR3 PE=1 SV=2	CFHR3	1	0	0	0	0	0	16,7	0,17	37,323	4,47E-03
P04083	Annexin A1 OS=Homo sapiens GN=ANXA1 PE=1 SV=2	ANXA1	0	0	0	1	0	0	16,7	0,17	38,714	4,31E-03
Q8N987	N-terminal EF-hand calcium-binding protein 1 OS=Homo sapiens GN=NECAB1 PE=1 SV	NECAB1	0	1	0	0	0	0	16,7	0,17	40,571	4,11E-03
P08476	Inhibin beta A chain OS=Homo sapiens GN=INHBA PE=1 SV=2	INHBA	0	0	1	0	0	0	16,7	0,17	47,442	3,51E-03
P04350	Tubulin beta-4A chain OS=Homo sapiens GN=TUBB4A PE=1 SV=2	TUBB4A	0	0	0	1	0	0	16,7	0,17	49,586	3,36E-03
Q3ZCM7	Tubulin beta-8 chain OS=Homo sapiens GN=TUBB8 PE=1 SV=2	TUBB8	0	0	0	1	0	0	16,7	0,17	49,776	3,35E-03
Q9BUF5	Tubulin beta-6 chain OS=Homo sapiens GN=TUBB6 PE=1 SV=1	TUBB6	0	0	0	1	0	0	16,7	0,17	49,857	3,34E-03
P13489	Ribonuclease inhibitor OS=Homo sapiens GN=RNH1 PE=1 SV=2	RNH1	0	0	0	0	0	1	16,7	0,17	49,973	3,34E-03
Q9P2R7	Succinate--CoA ligase [ADP-forming] subunit beta, mitochondrial OS=Homo sapiens GN=	SUCLA2	1	0	0	0	0	0	16,7	0,17	50,317	3,31E-03
Q05639	Elongation factor 1-alpha 2 OS=Homo sapiens GN=EEF1A2 PE=1 SV=1	EEF1A2	0	0	0	0	1	0	16,7	0,17	50,470	3,30E-03
P01008	Antithrombin-III OS=Homo sapiens GN=SERPINC1 PE=1 SV=1	SERPINC1	0	0	0	0	0	1	16,7	0,17	52,602	3,17E-03
Q9NZP8	Complement C1r subcomponent-like protein OS=Homo sapiens GN=C1RL PE=1 SV=2	C1RL	0	0	0	1	0	0	16,7	0,17	53,498	3,12E-03
P53985	Monocarboxylate transporter 1 OS=Homo sapiens GN=SLC16A1 PE=1 SV=3	SLC16A1	1	0	0	0	0	0	16,7	0,17	53,944	3,09E-03
C9JIZ6	Prosaposin OS=Homo sapiens GN=PSAP PE=1 SV=2	PSAP	0	1	0	0	0	0	16,7	0,17	58,441	2,85E-03
P09603	Macrophage colony-stimulating factor 1 OS=Homo sapiens GN=CSF1 PE=1 SV=2	CSF1	0	0	0	0	0	1	16,7	0,17	60,179	2,77E-03
Q96PD5	N-acetylmuramoyl-L-alanine amidase OS=Homo sapiens GN=PGLYRP2 PE=1 SV=1	PGLYRP2	1	0	0	0	0	0	16,7	0,17	62,217	2,68E-03
Q08357	Sodium-dependent phosphate transporter 2 OS=Homo sapiens GN=SLC20A2 PE=1 SV=	SLC20A2	0	0	0	0	1	0	16,7	0,17	70,392	2,37E-03
Q96M02	Centrosomal protein C10orf90 OS=Homo sapiens GN=C10orf90 PE=2 SV=2	C10orf90	0	0	0	0	0	1	16,7	0,17	77,910	2,14E-03
P33991	DNA replication licensing factor MCM4 OS=Homo sapiens GN=MCM4 PE=1 SV=5	MCM4	0	1	0	0	0	0	16,7	0,17	96,558	1,73E-03
Q76M96	Coiled-coil domain-containing protein 80 OS=Homo sapiens GN=CCDC80 PE=1 SV=1	CCDC80	0	1	0	0	0	0	16,7	0,17	108,174	1,54E-03
Q6ZMN7	PDZ domain-containing RING finger protein 4 OS=Homo sapiens GN=PDZRN4 PE=2 SV	PDZRN4	0	0	1	0	0	0	16,7	0,17	117,103	1,42E-03
Q5T0N1	Cilia- and flagella-associated protein 70 OS=Homo sapiens GN=CFAP70 PE=2 SV=3	CFAP70	0	0	0	1	0	0	16,7	0,17	125,721	1,33E-03

Q6ZU80	Centrosomal protein of 128 kDa OS=Homo sapiens GN=CEP128 PE=1 SV=2	CEP128	0	0	1	0	0	0	16,7	0,17	128,015	1,30E-03
P14543	Nidogen-1 OS=Homo sapiens GN=NID1 PE=1 SV=3	NID1	0	1	0	0	0	0	16,7	0,17	136,377	1,22E-03
P08603	Complement factor H OS=Homo sapiens GN=CFH PE=1 SV=4	CFH	1	0	0	0	0	0	16,7	0,17	139,096	1,20E-03
Q562F6	Shugoshin 2 OS=Homo sapiens GN=SGO2 PE=1 SV=2	SGO2	0	0	1	0	0	0	16,7	0,17	144,739	1,15E-03
P39060	Collagen alpha-1(XVIII) chain OS=Homo sapiens GN=COL18A1 PE=1 SV=5	COL18A1	0	0	0	0	1	0	16,7	0,17	178,188	9,35E-04
E9PHH0	Methyl-CpG-binding domain protein 5 OS=Homo sapiens GN=MBD5 PE=1 SV=1	MBD5	1	0	0	0	0	0	16,7	0,17	183,683	9,07E-04
E7EX88	Aggrecan core protein OS=Homo sapiens GN=ACAN PE=1 SV=2	ACAN	0	0	1	0	0	0	16,7	0,17	261,329	6,38E-04
A2RRP1	Neuroblastoma-amplified sequence OS=Homo sapiens GN=NBAS PE=1 SV=2	NBAS	0	0	0	1	0	0	16,7	0,17	268,571	6,21E-04
Q7Z2Y8	Interferon-induced very large GTPase 1 OS=Homo sapiens GN=GVINP1 PE=2 SV=2	GVINP1	0	1	0	0	0	0	16,7	0,17	279,048	5,97E-04
Q86YZ3	Hornerin OS=Homo sapiens GN=HRNR PE=1 SV=2	HRNR	0	0	0	0	0	1	16,7	0,17	282,390	5,90E-04
Q9NR99	Matrix-remodeling-associated protein 5 OS=Homo sapiens GN=MXRA5 PE=2 SV=3	MXRA5	0	0	0	0	0	1	16,7	0,17	312,150	5,34E-04
Q01484	Ankyrin-2 OS=Homo sapiens GN=ANK2 PE=1 SV=4	ANK2	1	0	0	0	0	0	16,7	0,17	433,715	3,84E-04
